# Regulation of Mus81-Eme1 structure-specific endonuclease by Eme1 SUMO-binding and Rad3^ATR^ kinase is essential in the absence of Rqh1^BLM^ helicase

**DOI:** 10.1371/journal.pgen.1010165

**Published:** 2022-04-22

**Authors:** Cédric Giaccherini, Sarah Scaglione, Stéphane Coulon, Pierre-Marie Dehé, Pierre-Henri L. Gaillard

**Affiliations:** Centre de Recherche en Cancérologie de Marseille, CRCM, Inserm, CNRS, Aix-Marseille Université, Institut Paoli-Calmettes, Marseille, France; The University of North Carolina at Chapel Hill, UNITED STATES

## Abstract

The Mus81-Eme1 structure-specific endonuclease is crucial for the processing of DNA recombination and late replication intermediates. In fission yeast, stimulation of Mus81-Eme1 in response to DNA damage at the G2/M transition relies on Cdc2^CDK1^ and DNA damage checkpoint-dependent phosphorylation of Eme1 and is critical for chromosome stability in absence of the Rqh1^BLM^ helicase. Here we identify Rad3^ATR^ checkpoint kinase consensus phosphorylation sites and two SUMO interacting motifs (SIM) within a short N-terminal domain of Eme1 that is required for cell survival in absence of Rqh1^BLM^. We show that direct phosphorylation of Eme1 by Rad3^ATR^ is essential for catalytic stimulation of Mus81-Eme1. Chk1-mediated phosphorylation also contributes to the stimulation of Mus81-Eme1 when combined with phosphorylation of Eme1 by Rad3^ATR^. Both Rad3^ATR^- and Chk1-mediated phosphorylation of Eme1 as well as the SIMs are critical for cell fitness in absence of Rqh1^BLM^ and abrogating bimodal phosphorylation of Eme1 along with mutating the SIMs is incompatible with *rqh1Δ* cell viability. Our findings unravel an elaborate regulatory network that relies on the poorly structured N-terminal domain of Eme1 and which is essential for the vital functions Mus81-Eme1 fulfills in absence of Rqh1^BLM^.

## Author summary

Structure-Specific Endonucleases (SSEs) are DNA cutting enzymes that process structures that form during DNA replication, recombination, repair and transcription. Their activities need to be tightly controlled to avoid that unscheduled DNA cutting drives genome instability. The fission yeast SSE Mus81-Eme1 specialized in the processing of DNA structures that link chromosomes, undergoes timely hyperactivation just before chromosome segregation. This involves cell cycle-driven phosphorylation of Eme1, which primes the protein for further phosphorylation by the DNA damage checkpoint. Here we discover that Eme1 is phosphorylated by the DNA damage sensor kinase Rad3^ATR^ and demonstrate that this is essential for the stimulation of Mus81-Eme1. Phosphorylation by the downstream effector Chk1 kinase is also required for full-fledged stimulation of Mus81-Eme1 but requires that Eme1 is also phosphorylated by Rad3^ATR^. In parallel, we show that Eme1 binds the small SUMO protein that modulates the functions/destiny of proteins to which it is attached. Interestingly, we provide evidence that these SUMO-binding properties contribute to the control of Mus81-Eme1 only in part through modulation of its activity. The importance of these different regulatory layers is underscored by the fact that together they are essential for cell viability in absence of the Rqh1^BLM^ helicase, which is related to the BLM helicase defective in highly cancer prone Bloom syndrome patients.

## Introduction

Structure-specific DNA endonucleases are cornerstones in the proper execution of DNA replication, repair and recombination; yet they harbor the potential for causing genome instability. Controlling these enzymes is essential to ensure efficient processing of appropriate substrates while preventing counterproductive targeting of other similar DNA structures. The Mus81-Eme1 structure-specific endonuclease (SSE) emerged from early on as a key player in the processing of recombination intermediates that form during homology directed repair of two-ended double strand breaks or during the rescue of stalled or broken replication forks [1–7]. Over this last decade, control mechanisms of Mus81-Eme1 that are tightly linked to cell cycle progression have been identified.

In *Saccharomyces cerevisiae (S*. *cerevisiae)*, Mus81-Mms4^EME1^ is stimulated at the G2/M transition by Cdc5^PLK1^-, Cdc28^CDK1^- and Dbf4-dependent phosphorylation of Mms4^EME1^ [8–12]. In human cells, catalytic upregulation of MUS81-EME1 is driven by complex formation with the SLX4 nuclease scaffold. This regulation is mediated by direct interaction between MUS81 and SLX4, which is strongly stimulated at the G2/M transition by phosphorylation of SLX4 by CDK1 [13]. Reminiscent of what has been observed in *S*. *cerevisiae*, maximal processing of joint molecules such as Holliday junctions by MUS81-EME1 also correlates with hyperphosphorylation of EME1, possibly by CDK1 or PLK1 [11]. Whether phosphorylation of EME1 contributes to the stimulation of MUS81-EME1 in human cells remains to be formally demonstrated. These mechanisms ensure that joint molecules such as Holliday junctions, D-loops or replication intermediates at under-replicated loci are efficiently resolved in mitosis before chromosome segregation [14]. By restricting the catalytic stimulation of Mus81-Eme1 to late stages of the cell cycle these control mechanisms also ensure that joint molecules and replication intermediates get a chance to be processed by more conservative non-endonucleolytic mechanisms that rely on their unfolding by RecQ helicases such as the BLM helicase in human cells and its Sgs1 and Rqh1 orthologs in *Saccharomyces cerevisiae* (*S. cerevisiae*) and *Schizosaccharomyces pombe* (*S*. *pombe*), respectively. These temporal controls further prevent the accumulation of hyper-activated Mus81-Eme1 in S-phase and the risk of the unscheduled processing of replication intermediates. The importance of such control mechanisms is underscored by the marked genomic instability that is caused by the premature stimulation of Mus81-Mms4 in budding yeast cells that produce an Mms4 mutant that mimics a constitutively phosphorylated Mms4 protein [9]. Interestingly, SUMOylation and ubiquitination of Mms4 were recently shown to specifically target phosphorylated Mms4 for degradation by the proteasome, further ensuring that hyperactivation of Mus81-Mms4 is restricted to mitosis [15]. In human cells, SLX4-MUS81 complex formation induced by premature activation of CDK1 results in the unscheduled processing of replication intermediates genome wide and chromosome pulverization [13].

In *S*. *pombe*, upregulation of Mus81-Eme1 also relies on the phosphorylation of Eme1 by Cdc2^CDK1^ [16]. However, in contrast to what has been described in *S*. *cerevisiae*, phosphorylation of Eme1 by Cdc2^CDK1^ primes Eme1 for further DNA damage checkpoint-mediated phosphorylation in response to DNA damage. This elaborate control mechanism ensures that Mus81-Eme1 is rapidly hyperactivated in response to DNA damage in late G2 and mitosis and is critical to prevent gross chromosomal rearrangements in absence of the BLM-related helicase Rqh1^BLM^ [16].

To gain further insight into the molecular mechanisms involved in the control of Mus81-Eme1 in *S*. *pombe*, we undertook *in silico* analyses of a relatively short N-terminal domain of Eme1 that we found to be essential for cell viability in the absence of Rqh1^BLM^. We identified Rad3^ATR^ consensus phosphorylation sites and two SUMO interacting Motifs (SIM1 and SIM2) within that domain. We demonstrate that Eme1 is a direct substrate for Rad3^ATR^ both *in vitro* and *in vivo* and show that phosphorylation of Eme1 by Rad3^ATR^ plays a prominent role in the catalytic stimulation of Mus81-Eme1 in response to DNA damage. Full-fledged stimulation of Mus81-Eme1 also relies on phosphorylation of Eme1 by Chk1 but, apparently, only when it occurs in conjunction with Rad3^ATR^-mediated phosphorylation of Eme1. We provide genetic evidence that both Rad3^ATR^—and Chk1-mediated phosphorylation of Eme1 are independently critical for cell fitness in the absence of Rqh1^BLM^. Remarkably, Chk1-mediated phosphorylation of Eme1 is lost when SIM2 is mutated, while mutating SIM1 has no impact. Both SIMs are important for cell fitness in absence of Rqh1^BLM^, and abrogating phosphoregulation of Mus81-Eme1 and mutating SIM1 and SIM2 recapitulates the synthetic lethality observed by deleting the N-terminus of Eme1 in the absence of Rqh1^BLM^.

## Results

### Eme1 N-terminus is essential in absence of Rqh1^BLM^

**Fig 1:**
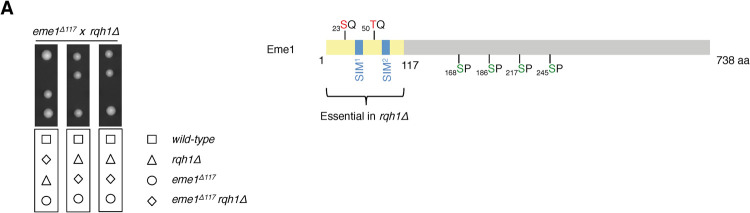
Eme1 N-terminal part (1–117) is essential in absence of Rqh1^BLM^. A- Tetrad analysis of an *eme1*^*Δ117*^ x *rqh1Δ* mating, germinated at 30°C. Boxes below dissections indicate the genotypes of each spore. B- Schematic of Eme1 protein. The yellow box depicts the first 117 amino-acid residues required for cell survival in absence of Rqh1^BLM^. The serine and threonine residues responding to Rad3^ATR^-consensus phosphorylation sites are depicted in red and the SUMO-Interacting Motifs are represented by blue squares. The serine residues targeted by Cdc2^CDK1^ are depicted in green.

To gain further insight into the mechanisms underlying the catalytic stimulation of Mus81-Eme1 in response to DNA damage, we searched for domains of Eme1 that are essential in absence of Rqh1^BLM^ while dispensable for the intrinsic catalytic activity of Mus81-Eme1. Interestingly, we found that deleting a relatively short N-terminal domain (residues 1–117) of Eme1 is synthetic lethal with *rqh1Δ* (**Fig 1A**). Importantly, this domain does not contain the Cdc2^CDK1^ sites that we had previously reported to be involved in the stimulation of Mus81-Eme1 in response to DNA damage and to be critical for cell viability in absence of Rqh1^BLM^ [16]. A detailed *in silico* analysis of the first 117 residues of Eme1 led to the identification of two SQ/TQ Rad3^ATR^ consensus sites (S_23_Q and T_50_Q) and two putative SUMO-Interacting Motifs (SIMs), hereafter named SIM1 and SIM2, which matched the described (V/I)-X-(V/I)-X-(V/I/L) consensus sequence (**Fig 1B**) [17]. These observations suggested that Eme1 might be a direct substrate for Rad3^ATR^ and possess SUMO-binding properties. We tested these predictions in the following experiments.

### Eme1 is a direct target of Rad3^ATR^ kinase

**Fig 2:**
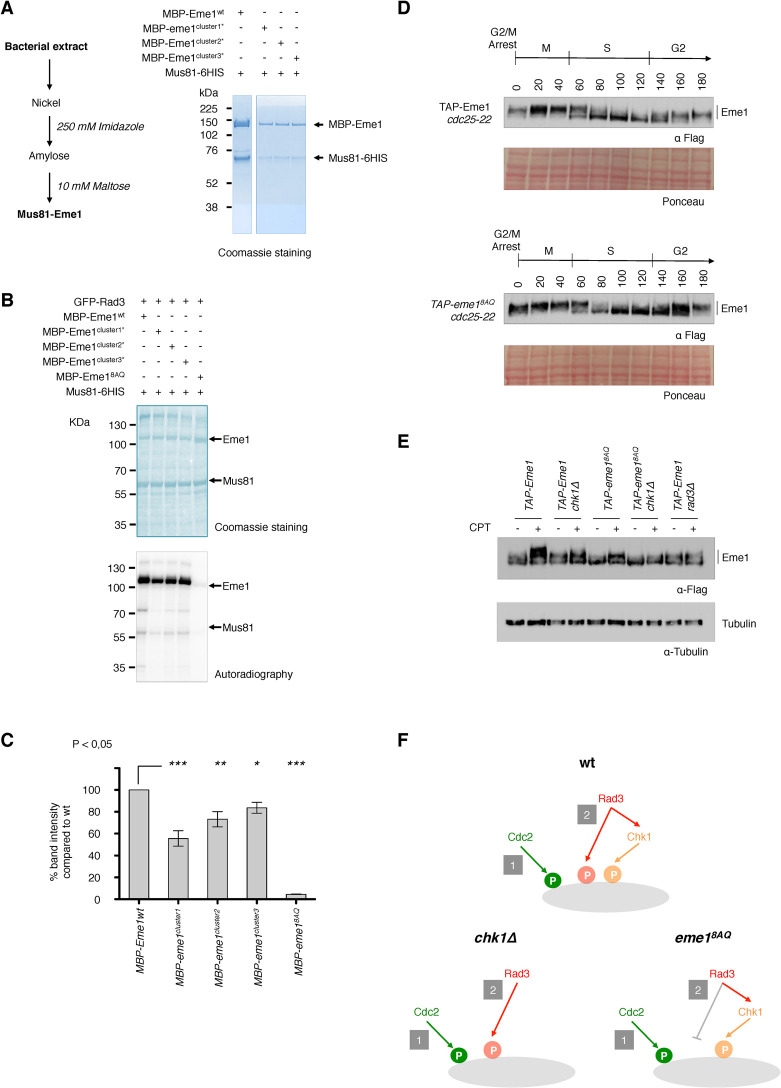
Rad3^ATR^ directly phosphorylates Eme1 *in vitro*. A- Recombinant *mus81* was co-expressed with either wild-type *eme1*, *eme1*^cluster1*^, *eme1*^cluster2*^ or *eme1*^cluster3*^ and purified from bacterial cultures (purification scheme is depicted on the left). Samples were loaded on an 4–12% Nupage Bis-Tris polyacrylamide gel alongside a Rainbow (Amersham) protein size marker. B- Representative autoradiography of ^32^P labeled Eme1. Rad3^ATR^ *in vitro* kinase assays were carried out on full-length recombinant Mu81-Eme1 complexes containing either wild-type Eme1 or Eme1 mutated for S_23_/T_50_ (cluster1*) or S_126_/T_145_/T_215_ (cluster2*) or S_313_/T_384_/S_458_ (cluster3*) or S_23_/T_50_/S_126_/T_145_/T_215_/S_313_/T_384_/S_458_ (eme1^8AQ^). Samples were loaded on a 3–8% Nupage Tris-Acetate polyacrylamide gel alongside a PageRuler (ThermoFisher) protein size marker. C- Relative band intensity of phosphorylated Eme1 (n = 3). All clusters contribute to Eme1 Rad3^ATR^-dependent phosphorylation with S_23_/T_50_ (cluster1*) being the most important. D- Cultures from *cdc25-22 TAP-eme1* and *cdc25-22 TAP-eme1*^*8AQ*^ were synchronized at the G2/M transition and released for one cell cycle. Total proteins were extracted at each indicated time point of the time course and analyzed by Western blot using an antibody raised against the Flag tag of Eme1. Ponceau stained membranes are depicted as loading control. E- Western blot on total lysates from untreated or 40 μM CPT–treated cells of the indicated background. Tubulin is used as a loading control. F- Scheme depicting the relative contributions of Cdc2^CDK1^, Rad3^ATR^ and Chk1 kinases in wild-type, *chk1Δ* and *eme1*^*8AQ*^ backgrounds. Note: TAP- = 2xProtA-TEVsite-2xFlag-.

Eme1 contains in total eight putative Rad3^ATR^ consensus phosphorylation sites (S_23_Q, T_50_Q, S_126_Q, T_145_Q, T_215_Q, S_313_Q, T_384_Q, S_458_Q) (**S1 Fig**). To determine whether Eme1 is a direct substrate of Rad3^ATR^ we set up *in vitro* kinase assays with recombinant Mus81-Eme1 and Rad3^ATR^. Recombinant Mus81(6His)-(MBP)Eme1 was produced in *E*. *coli* and affinity purified on Ni^++^ and amylose resins (**Fig 2A**). Recombinant (GFP)Rad3^ATR^ was instead transiently overproduced in yeast cells exposed to bleomycin to induce DNA damage and activate Rad3^ATR^. We used a *chk1Δ cds1Δ rad3Δ* mutant strain to eliminate the possibility of endogenous checkpoint kinases co-purifying with (GFP)Rad3^ATR^. As shown in **Fig 2B,** Eme1 was efficiently phosphorylated by (GFP)Rad3^ATR^. In contrast, an Eme1^8AQ^ mutant where all eight SQ/TQ sites are mutated to AQ is barely detected on the autoradiograph. To explore which SQ/TQ sites were important for phosphorylation we tested the impact of mutating Rad3^ATR^ phosphorylation sites in three different clusters (**S1 Fig**). The strongest effect was seen with mutations in Cluster 1, whilst mutations in Clusters 2 and 3 had a milder impact (**Fig 2B and 2C**). Noticeably, *in vitro* Rad3^ATR^-mediated phosphorylation of Eme1 does not require the priming by Cdc2^CDK1^. Taken together, these data indicate that the S_23_Q and T_50_Q sites in the N-terminus of Eme1 are most critical for its *in vitro* phosphorylation by Rad3^ATR^.

To investigate whether Eme1 is a substrate for Rad3^ATR^ *in vivo*, we generated an *eme1*^*8AQ*^ mutant strain in which all SQ/TQ sites are mutated to AQ (**S1 Fig**). As expected, whereas the cell-cycle dependent phosphorylation profile of Eme1^8AQ^ is comparable to that of the WT protein (**Fig 2D**), we observed a strong reduction of the CPT-induced phosphorylation (**Fig 2E**). However, DNA damage-dependent phosphorylation was not totally abolished in the Eme1^8AQ^ background (**Fig 2E**). We suspected that this residual phosphorylation of Eme1^8AQ^ was catalyzed by Chk1 (**Fig 2F**). Accordingly, we observed a complete loss of CPT-induced phosphorylation of Eme1^8AQ^ in *eme1*^*8AQ*^ *chk1Δ* cells (**Fig 2E**).

Overall, our data strongly indicate that Eme1 is phosphorylated by both Chk1 and Rad3^ATR^ following activation of the DNA damage checkpoint.

### Phosphorylation of Eme1 by Rad3^ATR^ is crucial in absence of Rqh1^BLM^

We previously showed that *in vivo* DNA damage-induced hyperphosphorylation of Eme1 is strictly subordinated to prior Cdc2^CDK1^-dependent phosphorylation [16]. Accordingly, mutating four CDK consensus target sites totally abrogates not only the cell-cycle dependent phosphorylation of the resulting Eme1^4SA^ protein but also its phosphorylation in response to DNA damage [16]. Importantly, the same study found that while an *eme1*^*4SA*^ single mutant displays no abnormal phenotype, an *eme1*^*4SA*^ *rqh1Δ* double mutant is extremely sick. These observations suggest that the Cdc2^CDK1^-dependent phosphorylations of Eme1 are required for its phosphorylation by both Rad3^ATR^ and Chk1, and that these events are critical in the absence of Rqh1^BLM^.

**Fig 3:**
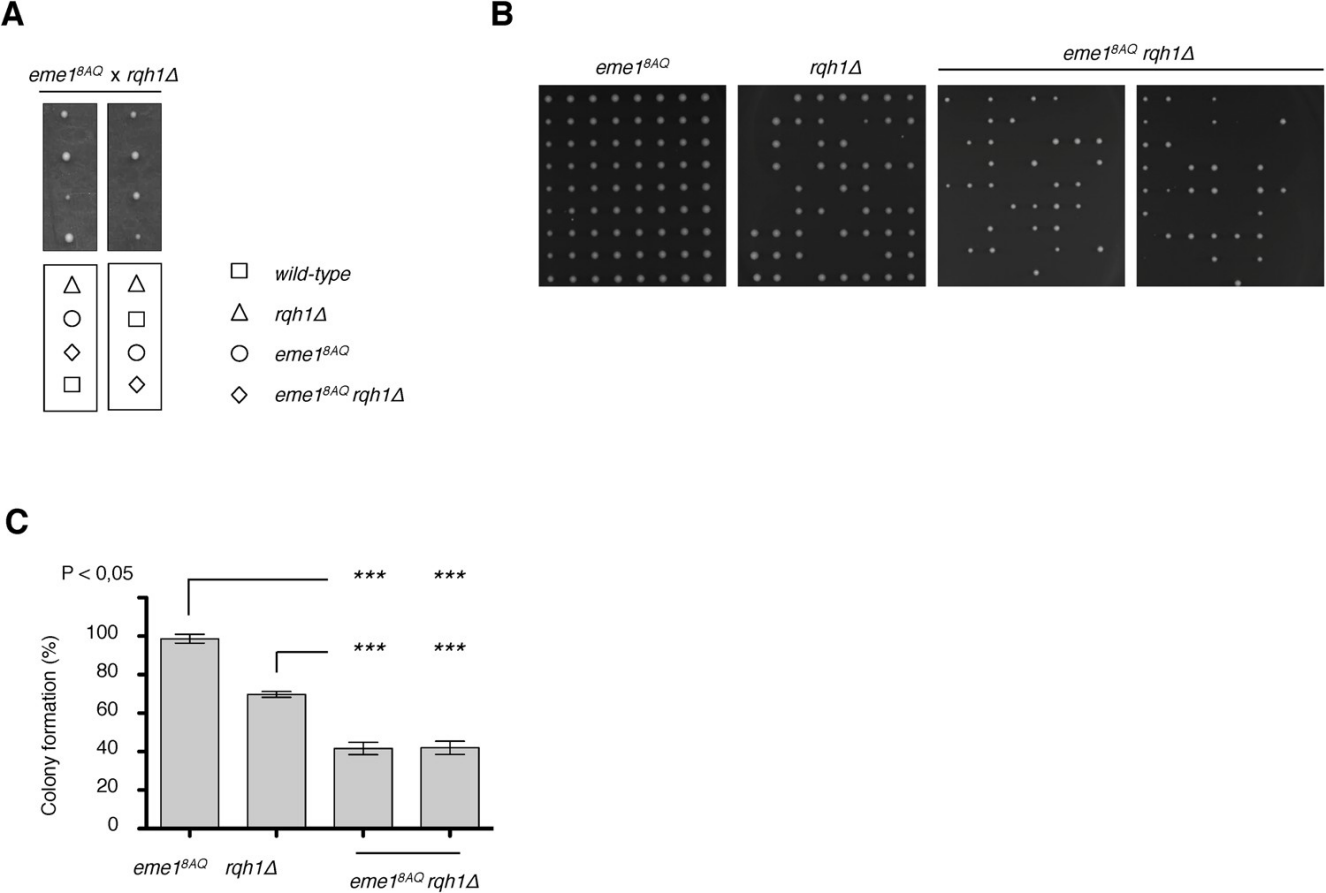
Negative genetic interaction between *eme1*^*8AQ*^ and *rqh1Δ*. A- Tetrad analysis of an *eme1*^*8AQ*^ x *rqh1Δ* mating, germinated at 30°C. Boxes below dissections indicate the genotypes of each spore. B- Exponentially growing *eme1*^*8AQ*^, *rqh1Δ* and *eme1*^*8AQ*^ *rqh1Δ* cells were seeded on YES plates by micromanipulation and allowed to grow for 3 days at 32°C. C- Average percentage (± s.d.) of viable colonies (n = 3 independent experiments). Statistical significance is measured with one-way ANOVA followed by Tuckey post-test.

To investigate the functional relevance of phosphorylation of Eme1 by Rad3^ATR^, we introduced *eme1*^*8AQ*^ mutations in the *rqh1Δ* background. While we observed no obvious phenotype for the *eme1*^*8AQ*^ single mutant, the *eme1*^*8AQ*^ *rqh1Δ* double mutant displayed pronounced growth and colony formation defects compared to the *rqh1Δ* single mutant (**Figs 3A, 3B, 3C and S2A**). This genetic interaction was further exacerbated by exposure to genotoxic agents (**S2B Fig**). Noteworthy, introducing the Eme1 SQ/TQ cluster mutations in the *rqh1Δ* background resulted in smaller colonies compared to the parental cells (**S2C Fig**). In line with the prominent contribution of Cluster 1 to Rad3^ATR^-mediated phosphorylation of Eme1 *in vitro* (**Fig 2B and 2C**), we noticed a more pronounced effect of Cluster 1 mutations on colony size in absence of Rqh1^BLM^ compared to the other two cluster mutations (**S2C Fig**). Overall, our data indicate that direct phosphorylation of Eme1 by Rad3^ATR^ is important for cell viability in the absence of Rqh1^BLM^.

### Rad3^ATR^ direct phosphorylation of Eme1 contributes to the catalytic stimulation of Mus81-Eme1

We have previously shown that Rad3^ATR^ contributes to the catalytic stimulation of the HJ-resolvase activity of Mus81-Eme1 [16]. We inferred at that time that the Rad3^ATR^-Chk1 axis was involved in this catalytic control. The finding that Rad3^ATR^ can directly phosphorylate Eme1, and that this phosphorylation is crucial in absence of Rqh1^BLM^, prompted us to investigate whether it contributed to the catalytic stimulation of Mus81-Eme1.

**Fig 4:**
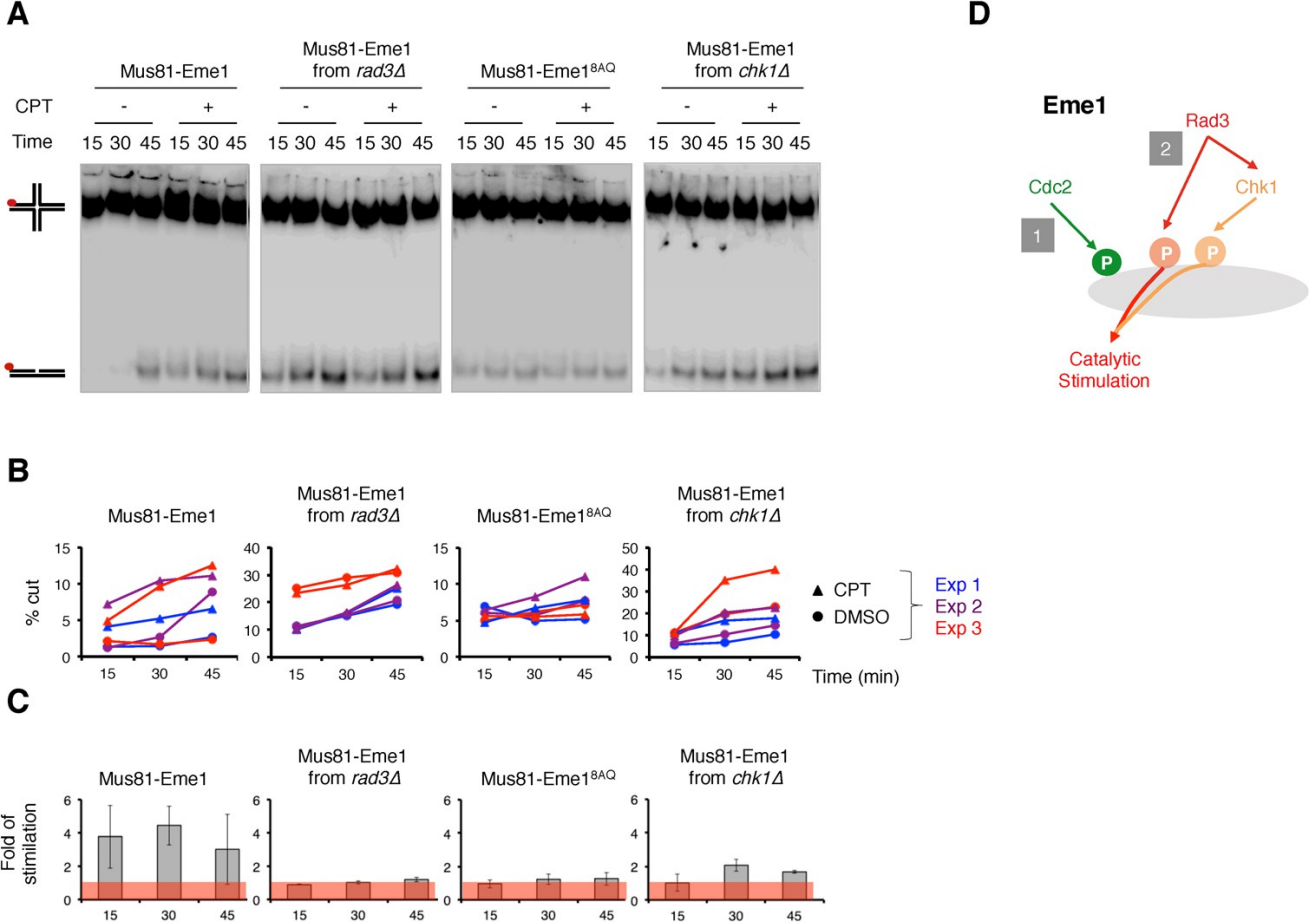
Rad3^ATR^ phosphorylation of Eme1 contributes to the catalytic stimulation of Mus81-Eme1 resolvase activity. A- ^32^P-labeled (red dot) HJs were incubated for the indicated times with wild type Mus81–Eme1 or mutant Mus81-Eme1^8AQ^ complexes recovered from untreated or 40 μM CPT–treated WT, *rad3Δ*, *eme1*^*8AQ*^ or *chk1Δ* mutant cells as described in Materials and Methods. Reaction products were analyzed by neutral PAGE. Comparable amounts of protein samples were used in each reaction after normalization of their relative concentration (see Materials and Methods and **S3A Fig**). B- Quantification of product formation, represented as a percentage of total radiolabeled DNA, in three independent experiments including the one in (A) (See **S3A, S3B, S3C, S3D and S3E Fig**). C- Average (± s.d.) fold stimulation of HJ resolution by Mus81–Eme1 following CPT–treatment (n = 3 independent experiments, see **S3 Fig**). The histogram shows the ratio of the HJ-resolvase activity of Mus81–Eme1 from CPT-treated cells over that of Mus81–Eme1 from untreated cells. D- Model for the Cdc2^CDK1^-, Rad3^ATR^- and Rad3^ATR^-Chk1-dependent phosphorylation of Eme1 and DNA damage-induced catalytic stimulation.

As previously reported [16], hyperphosphorylation of Eme1 following activation of the DNA damage checkpoint by CPT correlates with increased HJ-resolvase activity of Mus81-Eme1 complex isolated from fission yeast cells expressing TAP-tagged Eme1 (**Figs 4A, 4B, 4C, S3A and S3B**). Accordingly, no catalytic stimulation is detected when Mus81-Eme1 is recovered from *rad3Δ* cells ([16] and **Figs 4A, 4B, 4C, S3A and S3C**). Remarkably, we find that the Mus81-Eme1^8AQ^ complex is not stimulated following CPT treatment despite a fully functional DNA damage checkpoint and Chk1-mediated phosphorylation of Eme1 in *eme1*^*8AQ*^ cells (**Figs 2E, 4A, 4B, 4C, S3A and S3D**). In contrast to the complete loss of catalytic stimulation of Mus81-Eme1 in absence of Rad3^ATR^, some degree of catalytic stimulation of Mus81-Eme1 remains detectable in *chk1Δ* cells following CPT treatment (**Figs 4A, 4B, 4C, S3A and S3E**). Taken together, these data demonstrate that full-fledged catalytic stimulation of Mus81-Eme1 relies on phosphorylation of Eme1 by both Rad3^ATR^ and Chk1 kinases (**Fig 4D**). They also suggest that Chk1-mediated phosphorylation contributes to full activation of Mus81-Eme1 only when combined with phosphorylation of Eme1 by Rad3^ATR^.

### Eme1 contains bona-fide SIMs

**Fig 5:**
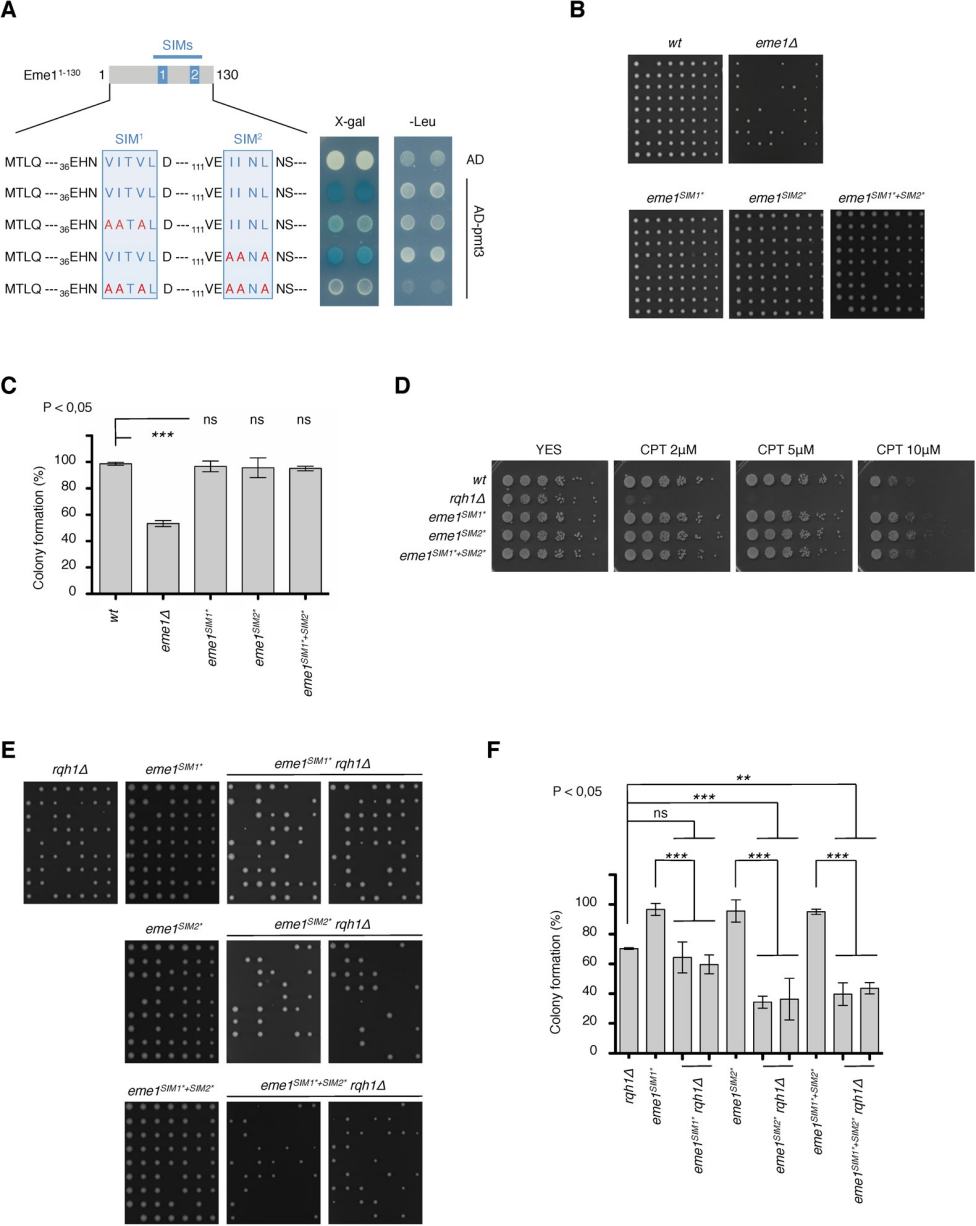
Eme1 contains *bona-fide* SIMs. A- A fragment of Eme1 (eme1^1-130^) containing SIM1 and SIM2 was used as bait in a Yeast two-hybrid assay for interaction with SUMO^PMT3^. The SIMs consensus sequences are indicated as well as the mutations introduced in each of them. B- Exponentially growing wild type, *eme1Δ*, *eme1*^SIM1*^, *eme1*^SIM2*^ and *eme1*^SIM1*+SIM2*^ cells were seeded on YES plates by micromanipulation and allowed to grow for 3 days at 32°C. C- Average percentage (± s.d.) of viable colonies (n = 3 independent experiments). Statistical significance is measured with one-way ANOVA followed by Tuckey post-test. D- Five-fold dilutions of wild-type, *rqh1Δ*, *eme1*^SIM1*^, *eme1*^SIM2*^ and *eme1*^SIM1*+SIM2*^ cells were plated on medium supplemented or not with the indicated concentrations of CPT followed by incubation at 30°C. E- Exponentially growing *rqh1Δ*, *eme1*^SIM1*^, *eme1*^SIM2*^ and *eme1*^SIM1*+SIM2*^ as well as two independent clones of *eme1*^SIM1*^ *rqh1Δ*, *eme1*^SIM2*^ *rqh1Δ* and *eme1*^SIM1*+SIM2*^ *rqh1Δ* were seeded on YES plates by micromanipulation and allowed to grow for 3 days at 32°C. F- Average percentage (± s.d.) of viable colonies (n = 3 independent experiments). Statistical significance is measured with one-way ANOVA followed by Tuckey post-test.

Having confirmed our predictions that Eme1 is directly phosphorylated by Rad3^ATR^ and shown that this is critical for catalytic stimulation of Mus81-Eme1 and cell fitness in absence of Rqh1^BLM^, we next undertook the analysis of the predicted SUMO-binding properties of Eme1 mediated by the putative SIM1 and SIM2 motifs in the N-terminal domain of Eme1 (**Figs 1B and 5A**).

The SUMO-binding capacities of SIM1 and SIM2 were assessed by a yeast two-hybrid assay against the unique *S*. *pombe* SUMO ortholog Pmt3. A fragment of Eme1 (Eme1^1-130^) containing SIM1 and SIM2 displayed strong binding to Pmt3, confirming that the N-terminus of Eme1 possesses SUMO-binding properties (**Fig 5A**). Introducing point mutations in the conserved aliphatic residues of SIM1 strongly impaired interaction with Pmt3 while mutations in SIM2 had a milder effect (**Fig 5A**). Mutations in both SIMs led to complete loss of interaction with Pmt3 (**Fig 5A**). Our data confirm that the N-terminal domain of Eme1, which is essential for cell viability in absence of Rqh1^BLM^ (**Fig 1A**), contains *bona fide* SIMs that jointly contribute to the SUMO-binding properties of Eme1, with a predominant contribution made by SIM1.

### Eme1 SIMs are required in absence of Rqh1^BLM^

To assess the functional relevance of SIM1 and SIM2, we generated mutant strains harboring the point mutations, described in **Fig 5A,** in SIM1 (*eme1*^SIM1*^), SIM2 (*eme1*^SIM2*^) or both SIMs (*eme1*^SIM1*+SIM2*^) of Eme1. None of the three *eme1*^SIM1*^, *eme1*^SIM2*^ and *eme1*^SIM1*+SIM2*^ mutant strains presented any obvious growth defect or reduced fitness compared to a WT strain in absence of exogenous DNA damage (**Fig 5B and 5C**) and following CPT treatment (**Fig 5D**). Since *eme1*^*Δ117*^ *rqh1Δ* double mutants are non-viable (**Fig 1A**), we next assessed the importance of SIM1 and/or SIM2 for cell viability in absence of Rqh1^BLM^. As shown in **Fig 5E and 5F**, while mutating SIM1 in the *rqh1Δ* background does not reduce colony formation capacities of the resulting *eme1*^SIM1*^ *rqh1Δ* double mutant compared to the *rqh1Δ* single mutant, it leads to a marked increase in the proportion of elongated and sick cells (**S4A Fig**). In contrast, mutating SIM2 strongly impairs the ability of *eme1*^SIM2*^ *rqh1Δ* to form viable colonies (**Fig 5E and 5F**) in addition to causing a strong increase in the number of sick cells (**S4A Fig**). Simultaneously mutating both SIMs did not further impair colony formation capacities compared to *eme1*^SIM2*^ *rqh1Δ* cells (**Fig 5E and 5F**). However, it had an additive effect regarding the proportion of elongated and sick cells (**S4A Fig**).

We further looked at cell fitness following chronic exposure to CPT. Loss of Rqh1^BLM^ in e*me1*^SIM1*^ mutant slightly exacerbated CPT sensitivity compared to *rqh1Δ*. This effect was slightly more pronounced for *eme1*^SIM2*^ *rqh1Δ* mutants while the *eme1*^SIM1*+SIM2*^ *rqh1Δ* mutants displayed the steepest increase in CPT sensitivity compared to *rqh1Δ* (**S4B Fig**). Overall, these data demonstrate that both SIMs contribute to the essential role of the Eme1^1-117^ N-terminal domain in absence of Rqh1^BLM^, with a more prominent contribution made by SIM2.

### Contribution of SIM1 and SIM2 to Eme1 phosphorylation and Mus81-Eme1 stimulation

**Fig 6:**
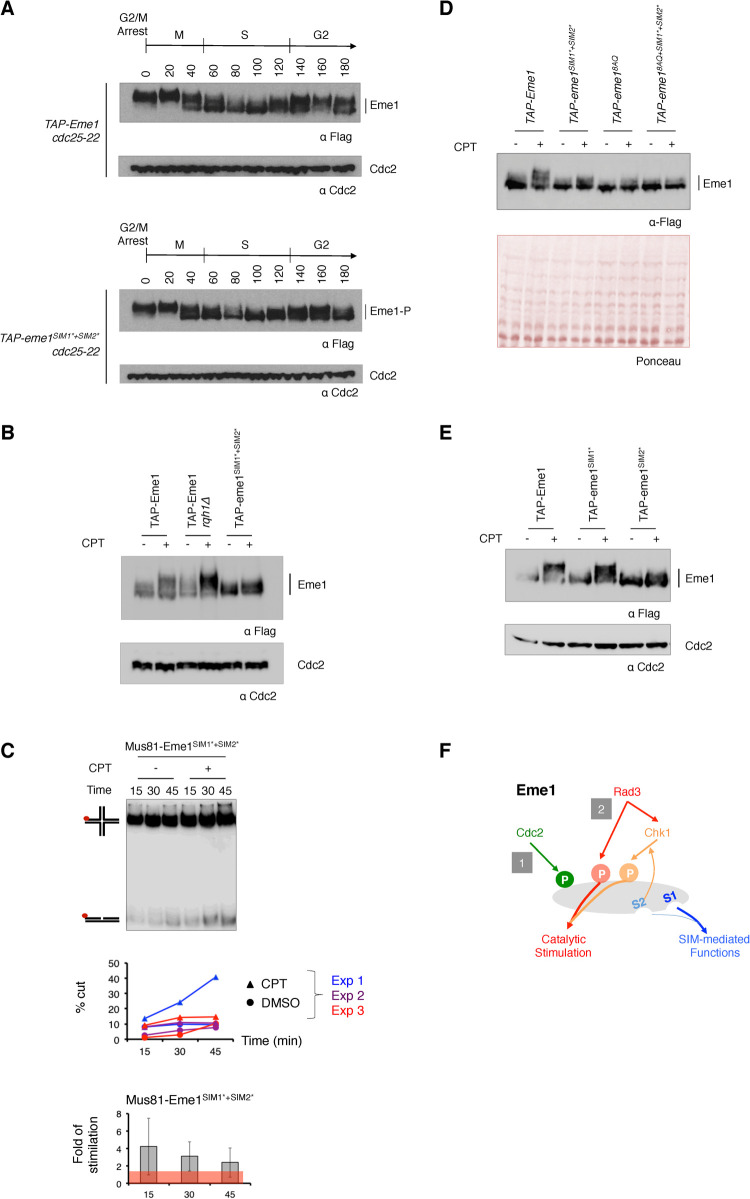
Mutations within the SIMs differently affect Eme1 phosphorylation. A- Western blot detection of TAP-Eme1 from *cdc25-22 TAP-eme1* and *cdc25-22 TAP- eme1*^SIM1*+SIM2*^ synchronized at the G2/M transition and released for one cell cycle. Total proteins were extracted at each indicated time point of the time course and analyzed by Western blot using an antibody raised against the Flag tag of TAP-Eme1. Cdc2 is used as a loading control. B- Western blot detection of TAP-Eme1 from untreated or 40 μM CPT–treated WT, *rqh1Δ* and *eme1*^SIM1*+SIM2*^ cells. Cdc2 is used as a loading control. C- ^32^P-labeled (red dot) HJs were incubated for the indicated times with Mus81-Eme1^SIM1*+SIM2*^ complex recovered from untreated or 40 μM CPT–treated *eme1*^SIM1*+SIM2*^ cells as described in Materials and Methods. Reaction products were analyzed by neutral PAGE. Comparable amounts of protein samples were used in each reaction after normalization of their relative concentration (see Materials and Methods and **S3A Fig**). A first graph below the gel represents the quantification of product formation, as a percentage of total radiolabeled DNA, in three independent experiments including the one shown above the graph (See **S3A and S5 Figs**). A second graph shows the average (± s.d.) fold stimulation of HJ resolution by Mus81-Eme1^SIM1*+SIM2*^ following CPT–treatment (n = 3 independent experiments, see **S5 Fig**). The histogram shows the ratio of the HJ-resolvase activity of Mus81–Eme1 from CPT-treated cells over that of Mus81–Eme1 from untreated cells. D- Western blot detection of TAP-Eme1 from untreated or 40 μM CPT–treated WT, *eme1*^SIM1*+SIM2*^, *eme1*^*8AQ*^ and *eme1*^8AQ+SIM1*+SIM2*^ cells. Ponceau stained membranes are depicted as loading control. E- Western blot detection of TAP-Eme1 from untreated or 40 μM CPT–treated WT, *eme1*^SIM1*^ and *eme1*^SIM2*^ cells. Cdc2 is used as a loading control. F- Model summarizing our findings on the relative contributions of Rad3^ATR^, Chk1 and the SIMs to phosphorylation of Eme1 and catalytic stimulation of Mus81-Eme1 in response to DNA damage. Note: TAP- = 2xProtA-TEVsite-2xFlag-.

The importance of the Eme1 SIMs in absence of Rqh1^BLM^ prompted us to assess possible functional ties between the SUMO-binding properties of Eme1 and its phosphorylation. Interestingly, whereas mutating the SIMs had no obvious effect on the phosphorylation profile of Eme1^SIM1*+SIM2*^ throughout the cell cycle (**Fig 6A**), it substantially reduced phosphorylation levels in response to CPT (**Fig 6B**). It also reduced the catalytic stimulation of the Mus81-Eme1^SIM1*+SIM2*^ complex, compared to the WT complex, following CPT treatment (**Figs 6C, S3A and S5**). This suggested that mutating the SIMs did not abrogate phosphorylation of Eme1 by Rad3^ATR^, which we showed results in complete loss of DNA damage induced stimulation of Mus81-Eme1 (**Figs 4A, 4B, 4C, S3A, S3C and S3D**). In line with this, Eme1^SIM1*+SIM2*^ is efficiently phosphorylated by Rad3^ATR^ *in vitro* (**S6 Fig**). To assess whether the residual CPT-induced mobility shift of Eme1^SIM1*+SIM2*^ is due to phosphorylation by Rad3^ATR^ *in vivo*, we generated an *eme1*^8AQ+SIM1*+SIM2*^ mutant. Mutating all Rad3^ATR^ consensus phosphorylation sites and both SIMs resulted in the complete loss of CPT-induced mobility shift of Eme1 (**Fig 6D**), confirming that Eme1^SIM1*+SIM2*^ remains phosphorylated by Rad3^ATR^ in response to DNA damage. These data suggest that the SIMs promote Chk1-mediated phosphorylation of Eme1 and as such contribute to the catalytic stimulation of Mus81-Eme1, which is critical for cell fitness in absence of Rqh1^BLM^ [16]. To gain further insight into the respective contribution of SIM1 and SIM2 to Chk1-mediated phosphorylation of Eme1 we compared the CPT-induced mobility shifts of Eme1^SIM1*^ and Eme1^SIM2*^ mutants (**Fig 6E**). While mutating SIM1 had barely any impact, mutating SIM2 substantially reduced phosphorylation levels in response to CPT (**Fig 6E**). Based on these findings and the severe growth defect of an *eme1*^SIM2*^ *rqh1Δ* double mutant (**Figs 5E, 5F and S4A**), we propose that phosphorylation of Eme1 by Chk1, promoted by SIM2, represents an additional phosphorylation-based layer of control of Mus81-Eme1 that is critical for cell viability in absence of Rqh1^BLM^ (**Fig 6F**).

### SUMO-binding capacities and phosphorylation cooperate for cell viability in absence of Rqh1^BLM^

**Fig 7:**
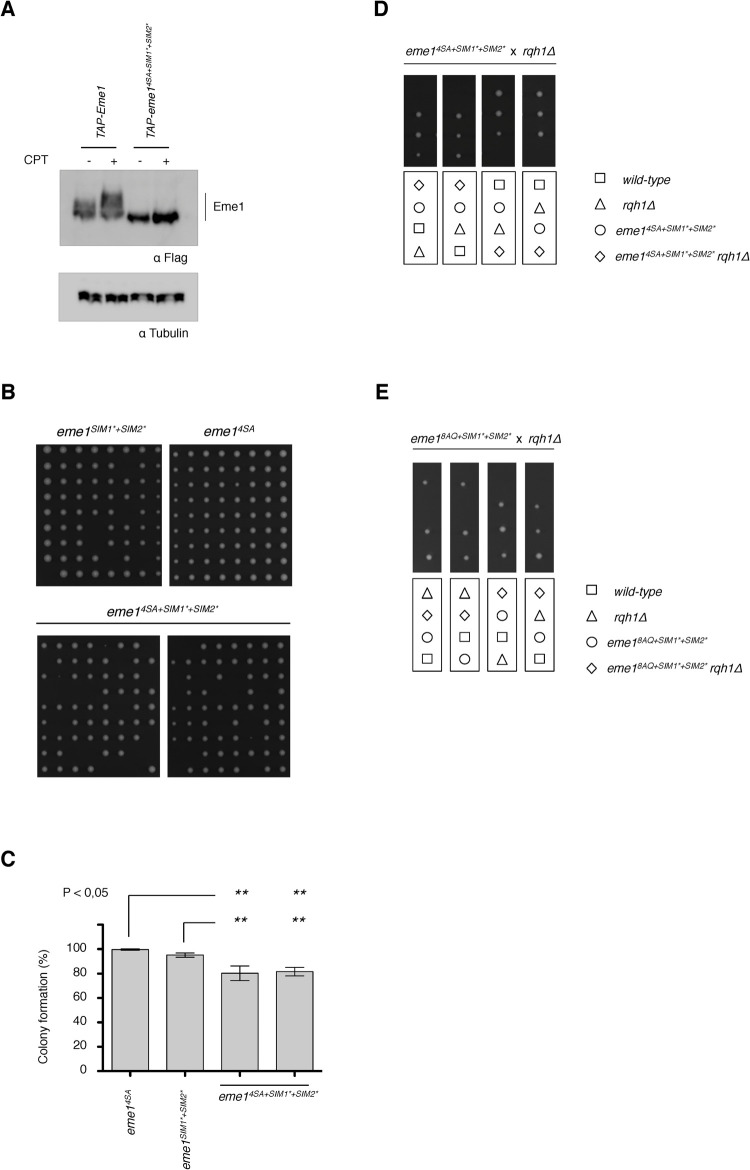
The combined loss of Eme1 phosphorylation and SUMO-binding properties is lethal in absence of Rqh1^BLM^. A- Western blot detection of TAP-Eme1 on total lysates from untreated or 40 μM CPT–treated from WT or *eme1*^4SA+SIM1*+SIM2*^ cells. An Eme1^4SA+SIM1*+SIM2*^ protein shows no constitutive Cdc2^CDK1^-dependent (-CPT) and DNA damage-induced mobility shift (+CPT). Tubulin is used as a loading control. Note: TAP- = 2xProtA-TEVsite-2xFlag- B- Exponentially growing *eme1*^SIM1*+SIM2*^, *eme1*^*4SA*^ and *eme1*^4SA+SIM1*+SIM2*^ cells were seeded on YES plates by micromanipulation and allowed to grow for 3 days at 32°C. C- Average percentage (± s.d.) of viable colonies (n = 3 independent experiments). Statistical significance is measured with one-way ANOVA followed by Tuckey post-test. D- Tetrad analysis of an *eme1*^4SA+SIM1*+SIM2*^ x *rqh1Δ* mating, germinated at 30°C. Boxes below dissections indicate the genotypes of each spore. E- Tetrad analysis of an *eme1*^8AQ+SIM1*+SIM2*^ x *rqh1Δ* mating, germinated at 30°C. Boxes below dissections indicate the genotypes of each spore.

**Fig 8:**
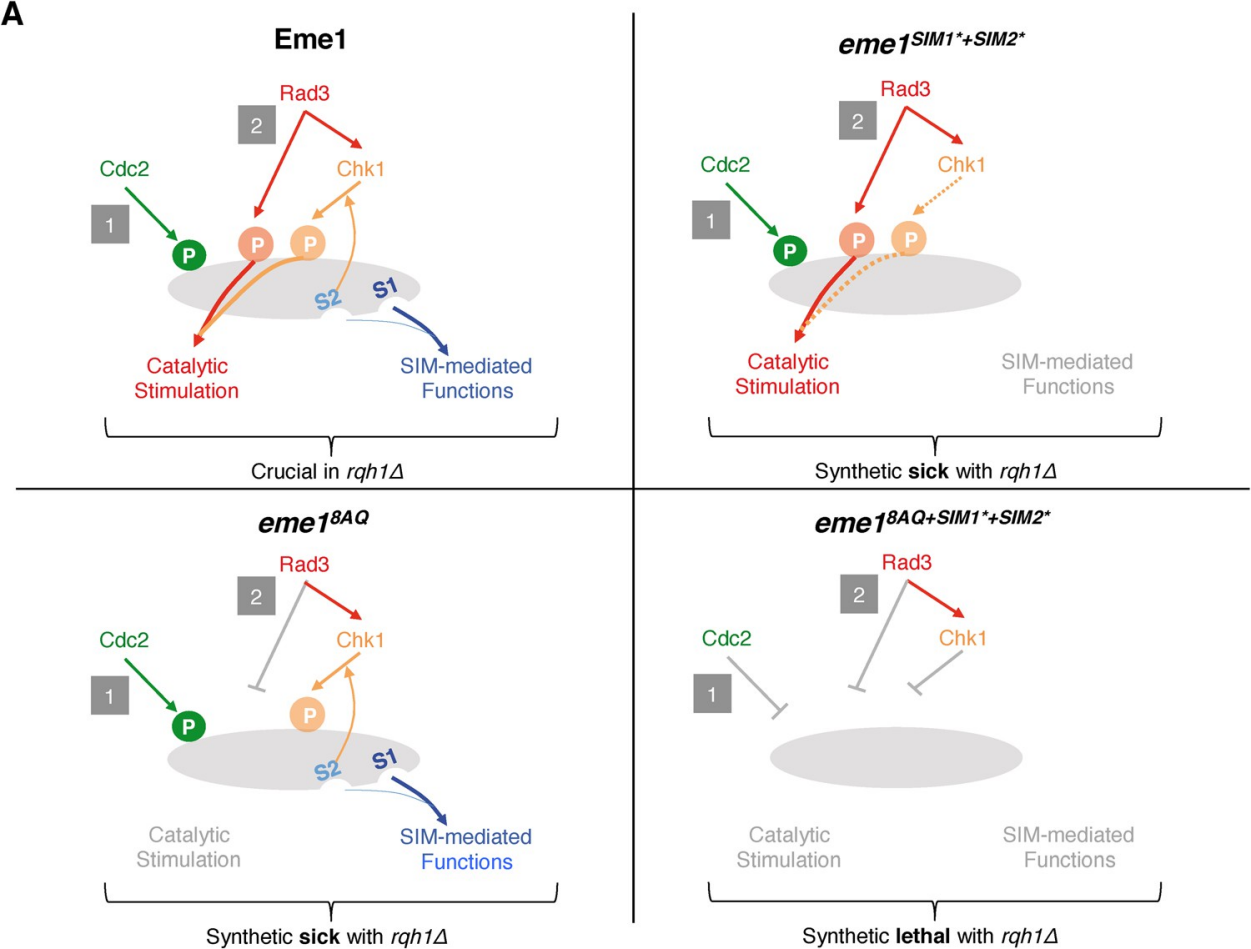
Model for the synthetic lethality resulting from combined loss of Eme1 phosphorylation and SUMO-binding properties in absence of Rqh1^BLM^. See text for details.

To further assess whether we have unraveled layers of regulation of Mus81-Eme1 that all contribute in their own way to cell survival in absence of Rqh1^BLM^, we undertook genetic analyses by combining the mutations that impair the SIMs (i.e. *eme1*^SIM1*+SIM2*^) with those that abrogate the cell cycle and DNA damage-dependent phosphorylations of Eme1 (i.e. *eme1*^*4SA*^). As expected, we observed no constitutive Cdc2^CDK1^-dependent and DNA damage-induced mobility shift of the Eme1^4SA+SIM1*+SIM2*^ mutant protein (**Fig 7A**). The resulting *eme1*^4SA+SIM1*+SIM2*^ mutant strain displays a slightly reduced ability to form viable colonies in absence of exogenous stress (**Fig 7B and 7C**). Remarkably, while *eme1*^SIM1*+SIM2*^ *rqh1Δ* (**Fig 5E**) and *eme1*^*4SA*^ *rqh1Δ* [16] double mutant strains are sick but viable, we were unable to generate viable *eme1*^4SA+SIM1*+SIM2*^ *rqh1Δ* cells (**Fig 7D**). We next crossed *eme1*^8AQ+SIM1*+SIM2*^ and *rqh1Δ* cells to determine to which extent direct phosphorylation of Eme1 by Rad3^ATR^ and the SIM related functions (including phosphorylation by Chk1) contribute to the essential functions of Mus81-Eme1 in absence of Rqh1^BLM^. As shown in **Fig 7E**, we were also unable to recover *eme1*^8AQ+SIM1*+SIM2*^ *rqh1Δ* cells. This is reminiscent of the synthetic lethal interaction between *eme1*^*Δ117*^ and *rqh1Δ* and suggests that the Eme1^1-117^ N-terminal domain is involved in three regulatory processes that each make key contributions to the essential functions fulfilled by Mus81-Eme1 in absence of Rqh1^BLM^ (**Fig 8**).

## Discussion

In this study we have identified three layers of control of Mus81-Eme1 that are critical in absence of Rqh1^BLM^ (**Figs 6F and 8**). A first layer relies on the phosphorylation of Eme1 by Rad3^ATR^ in response to DNA damage and is critical for the catalytic stimulation of Mus81-Eme1 (**Figs 4D, 6F and 8**). Another layer that drives catalytic stimulation of Mus81-Eme1 relies on the phosphorylation of Eme1 in response to DNA damage by Chk1 (**Figs 4D, 6F and 8**). It is noteworthy that both these layers require that Eme1 is first phosphorylated by Cdc2^CDK1^ [16]. The third layer relies on newly described SIM1- and SIM2-dependent SUMO-binding properties of Eme1. Importantly, our results reveal that there is some crosstalk between these layers with, on one hand, Chk1-mediated stimulation of Mus81-Eme1 requiring that Eme1 is also phosphorylated by Rad3^ATR^ and, on the other, SIM2 contributing to phosphorylation of Eme1 by Chk1 (**Figs 6F and 8**).

In our initial study we had suggested that catalytic stimulation of Mus81-Eme1 in response to DNA damage relied on the phosphorylation of Eme1 by a classical DNA damage checkpoint signaling mechanism and that it was ultimately driven by phosphorylation of Eme1 by Chk1 [16]. With the demonstration that DNA damage-induced stimulation of Mus81-Eme1 strictly relies on direct phosphorylation of Eme1 by Rad3^ATR^ (**Figs 4A, 4B, 4C, S3A and S3C**), we are providing new insight into a control mechanism of Mus81-Eme1 that turns out to be substantially more elaborate than initially anticipated.

Indeed, mutating all eight Rad3^ATR^ SQ/TQ phosphorylation sites in Eme1 prevents phosphorylation by Rad3^ATR^ *in vitro* and *in vivo* (**Fig 2B and 2E**) and fully abrogates DNA damage-induced stimulation of the resulting Mus81-Eme1^8AQ^ complex (**Figs 4A, 4B, 4C, S3A and S3D**), even though Eme1^8AQ^ still undergoes Chk1-mediated phosphorylation (**Fig 2E and 2F**). These data point towards an essential role of direct phosphorylation by Rad3^ATR^ and question whether phosphorylation by Chk1 makes any contribution at all to the catalytic stimulation of Mus81-Eme1. However, we also found that in absence of Chk1, Mus81-Eme1 is very poorly stimulated by CPT treatment even though Eme1 is still directly phosphorylated by Rad3^ATR^ (**Figs 4A, 4B, 4C, S3A and S3E**). Based on these findings, we propose a model whereby full-fledged stimulation of Mus81-Eme1 in fact relies on phosphorylation of Eme1 by both Rad3^ATR^ and Chk1 (**Figs 6F and 8**). The complete lack of stimulation of the Mus81-Eme1^8AQ^ complex, despite Chk1-mediated phosphorylation of Eme1^8AQ^ suggests that stimulation by Chk1 only occurs when Eme1 is also phosphorylated by Rad3^ATR^. However, we cannot formally exclude at this stage that the 8AQ mutations could have a knock-on effect that impairs phosphorylation by Chk1 (without any obvious impact on the mobility shift of Eme1).

It is noteworthy that all Rad3^ATR^ SQ/TQ phosphorylation sites are located before the coiled-coil domain of Eme1 and sit therefore within an intrinsically disordered part of Eme1. This is particularly relevant for the first two phosphorylation sites found in cluster 1 which are the only two within the Eme1^1-117^ domain. They are the ones that also contribute the most to the phosphorylation of Eme1 by Rad3^ATR^ *in vitro* and cell fitness in absence of Rqh1^BLM^ (**S2C Fig**). It will be important to further investigate what structural impact phosphorylation of the N-terminus of Eme1 may have or whether it drives conformational changes by promoting the association with a coactivator and how this stimulates Mus81-Eme1. Hints that the poorly structured N-terminal domain of Eme1 may negatively impact the catalytic activity of Mus81-Eme1 may be seen in the increased *in vitro* activity that results from clipping off large N-terminal domains of human MUS81 and EME1 that are dispensable for the endonuclease function of the complex [18]. This would also be reminiscent of the auto-inhibition of human MUS81-EME1 by the N-terminal Helix-hairpin-Helix domain within MUS81 that is relieved upon association of the N-terminus of MUS81 with SLX4 [19]. Cryo-EM studies of XPF-ERCC1 also revealed how conformational changes imposed by DNA binding relieved auto-inhibition by the N-terminal helicase domain of XPF [20]. Members of the XPF-family of SSEs, which all carry their catalytic-relevant functions in the C-terminal part of their subunits, appear to have evolved in a way that provides their N-terminal domains with regulatory functions mediated through controlled conformational changes. Based on our findings, such conformational changes driven by phosphorylation seem like a plausible explanation in the case of Eme1.

Another pivotal finding of this study is the identification of two SIMs (SIM1 and SIM2) in the Eme1^1-117^ N-terminal domain. Noteworthy, as observed for the *eme1*^*4SA*^ and *eme1*^*8AQ*^ phosphorylation mutants, mutating the SIMs of Eme1 severely impacts cell viability in absence of Rqh1^BLM^ (**Fig 5E and 5F**) but does not lead to any increased sensitivity to CPT (**Fig 5D**). This contrasts with the acute hypersensitivity of *mus81Δ* and *eme1Δ* null cells and indicates that the Eme1 SIM-dependent functions are intimately linked to the control of Mus81-Eme1 in relation to Rqh1^BLM^ functions.

We show that mutating the SIMs results in a marked decrease in DNA damage induced phosphorylation of Eme1 by Chk1 and tampers catalytic stimulation of Mus81-Eme1 (**Figs 6B, 6C, S3A and S5**). The drop in catalytic stimulation is less extensive than what might be expected from the pronounced reduction in DNA damage induced phosphorylation of Eme1. It is noteworthy that Eme1^SIM1*+SIM2*^ remains phosphorylated by Rad3^ATR^ and there remains the possibility that it undergoes residual Chk1-mediated phosphorylation that does not induce a detectable mobility shift but contributes to catalytic stimulation. Importantly, our genetic analyses indicate that the SIMs fulfil pivotal functions beyond catalytic control of Mus81-Eme1. Indeed, whereas Eme1 mutants unable to undergo catalytic stimulation (*eme1*^*4SA*^ or *eme1*^*8AQ*^) or to bind SUMO (*eme1*^SIM1*+SIM2*^) are viable in absence of Rqh1^BLM^ ([16], **Figs 3B and 5E**), Eme1 mutants that combine both defects (*eme1*^4SA+SIM1*+SIM2*^ and *eme1*^8AQ+SIM1*+SIM2*^) are not (**Fig 7D and 7E**). Such non-catalytic functions of the SIMs may drive the efficient recruitment and stabilization of Mus81-Eme1 at sites where it is needed to process secondary DNA structures that accumulate in absence of Rqh1^BLM^. In line with this, it was recently proposed that the human SLX4 nuclease scaffold that targets the XPF-ERCC1, MUS81-EME1 and SLX1 SSEs to specific genomic loci contains several SIMs that are involved in its own recruitment to telomeres, PML bodies and DNA damage [21–23]. An alternative and radically opposite explanation could be that the SIMs of Eme1 are involved in a process that negatively controls Mus81-Eme1 by sequestering the nuclease in subnuclear compartments, away from DNA secondary structures such as replication intermediates that could otherwise get opportunistically processed. In absence of Rqh1^BLM^ such structures would accumulate and their premature endonucleolytic processing would be deleterious to the cell. Such compartmentalisation has been observed in human cells with the nucleolar accumulation of MUS81-EME1 in S-phase and its relocalisation out of the nucleolus to sites of DNA damage in replicating cells following UV-irradiation [7].

Remarkably, while we find that SIM1 and SIM2 cooperate to bind SUMO (**Fig 5A**), we provide evidence that they also fulfil independent functions. First, mutating the strong SUMO binding SIM1 causes the accumulation of sick cells in absence of Rqh1^BLM^ but it barely impacts colony formation, whereas mutating the much weaker SUMO-binder SIM2 significantly reduces colony formation in addition to causing the accumulation of sick cells. Mutating both SIMs synergistically increased the proportion of sick cells (**Figs 5E, 5F and S4A**). Furthermore, we also found that SIM2 but not SIM1 promotes Chk1-dependent phosphorylation of Eme1 in response to DNA damage (**Fig 6E**). These results not only suggest that each SIM fulfils different functions, they also question whether some of those fulfilled by SIM2 might extend beyond SUMO-binding when putting into perspective the SIM2-specific phenotypes and its relatively poor affinity for SUMO compared to SIM1. In line with this, we found that Eme1 still undergoes DNA damage-triggered phosphorylation in mutant cells that lack the Pli1 and Nse2 SUMO E3 ligases or that do not produce SUMO (**S7 Fig**). At first glance, this might indicate that the processes that lead to the phosphorylation of Eme1 in response to DNA damage do not involve SUMO, including those that rely on SIM2. However, the possibility remains that in absence of SUMO, DNA damage-induced phosphorylation of Eme1 is entirely driven by Rad3^ATR^, which does not rely on Eme1 SIMs (**Fig 6D**). Further work is therefore needed to formally establish whether SIM2 promotes phosphorylation of Eme1 by Chk1 through interaction with SUMO or not. While we cannot exclude that the mutations introduced in SIM2 induce structural changes that impact more than just SUMO-binding, should SUMO not be involved there remains the exciting possibility that SIM2 could drive the association of Eme1 with a partner that contains a SUMO-like domain (SLD) [24]. The only SLD-containing protein described so far in *S*. *pombe* is the Rad60 genome stability factor that contains two SLDs, each of which interact with different players of the SUMO pathway [25]. Interestingly, the presumed *S*. *cerevisiae* Rad60 ortholog Esc2 has been reported to interact with Mus81 via its SLDs and to stimulate the Mus81-Mms4 complex [26]. In addition, Esc2 was recently found to promote the degradation of phosphorylated Mms4 [15]. We can exclude similar scenarios involving Rad60 as none of the SIMs were found to modulate the levels of Eme1 or phosphorylated Eme1. However, based on such functional promiscuity between Mus81-Mms4 and the SLD-containing Esc2 protein, it is tempting to see Rad60 as the ideal candidate for a SIM2-mediated partner of Eme1 that would promote Chk1-dependent phosphorylation of Eme1 in response to DNA damage. This would not be the first example of regulatory processes that involve similar players but different outcomes for Mus81-Eme1 and Mus81-Mms4.

Overall, our findings show that the poorly structured N-terminal domain of Eme1 harbors essential regulatory functions of Mus81-Eme1, the control of which appears to be remarkably more elaborate than initially described. With the demonstration that it relies on three regulatory layers that together contribute to the vital functions it fulfils in cells lacking Rqh1^BLM^, we are setting the basis for new lines of investigation that should contribute to a better understanding of the contributions made by Mus81-Eme1 in the maintenance of genome stability.

## Materials and methods

### Fission yeast strains, media, techniques and plasmids

Fission yeast strain genotypes are listed in **S1 Table**. Media and methods for studying *S*. *pombe* were as described elsewhere [27].

The *eme1* mutants (*eme1*^*8AQ*^, *eme1*^SIM1*^, *eme1*^SIM2*^, *eme1*^SIM1*+SIM2*^, *eme1*^SIM1*+SIM2*+4SA^) were generated as follows. The *Eme1* genomic locus from strain PH81 (*h*+, *leu1*-*32 ura4*-*D18 TAP-eme1 mus81*:*13Myc*-*KanMX6*), which produces N-terminally TAP-tagged Eme1, was subcloned into a TopoTA vector (Invitrogen). Point mutations were introduced on that TopoTA-*EME1* vector by using a Multiprime site-directed mutagenesis kit (Stratagene). Mutations were confirmed by DNA sequencing. The mutated *Eme1* genomic locus from the TopoTA-*Eme1* vector was used as template for PCR using primers forward 5’–acccatctctcacctaacc– 3’ and reverse 5’- cagtattagcttacagcc– 3’. The PCR fragment was then used to transform strain PH41, in which a URA4 cassette replaces the start codon of EME1 gene. 5′-FOA–resistant clones were selected and confirmed as *TAP-eme1* mutant producing strains by genomic DNA sequencing.

### Cell synchronization

For synchronization of cells by *cdc25-22* block and release, cells containing the temperature-sensitive *cdc25-22* allele were grown to exponential phase at permissive temperature (25°C) and shifted at restrictive temperature (36°C) for 3.5 h to arrest the cell cycle in G2. Upon release to permissive temperature (25°C), the cells synchronously enter the cell cycle. Cells were collected and processed every 20 min. Progression into S phase was monitored microscopically by counting cells that contained septa using calcofluor (Sigma) staining, the appearance of which correlates with S phase.

For DNA damage studies, Bleomycin (Merck) was added to cells arrested at the G2/M transition and further incubated at 36°C for 1,5h.

### Colony formation assay

Fresh cultures were re-seeded on YES plates by micromanipulation and allowed to grow for 3 days at 32°C. At least 3 independent experiments were performed and averaged. Statistical significance (1 way ANOVA and Tuckey test) is displayed on each graph.

### Yeast two-hybrid

Yeast strains were derived from EGY48 (*MAT*α, *ura3*, *his3*, *trp1*, and *LexA*_*op(x6)*_*-LEU2*) containing the pSH18-34 (*LexA*_*op(x8)*_*-LacZ*, *URA3*, and *amp*^*r*^) plasmid. The fission yeast complementary DNA (cDNA) coding for the first 130 amino acid was cloned into the pJG4-5 (*B42-AD*, *TRP1*, and *amp*^*r*^) and the pEG202 (*LexA*_*(1–202)*_*DNA-BD*, *HIS3*, and *amp*^*r*^) and cotransformed into EGY48 + pSH18-34 strain and plated onto -URA-TRP-HIS medium. To monitor protein interaction, clones were spotted onto 3% Gal-URA-TRP-HIS-LEU and 3% Gal-URA-TRP-HIS-Xgal (80 μg/ml) plates. Plates were incubated at 30°C for 2 to 4 days.

### Protein extraction, immunoprecipitation and immunoblotting

Cellular lysates were prepared from exponentially growing cell cultures treated with 40μM camptothecin (Sigma) or 5μg/ml bleomycin (Merck). Denatured cell lysates were prepared by TCA precipitation. Cells were suspended in 20% TCA and lysed mechanically using glass beads (Sigma). Following centrifugation, the TCA precipitate was suspended in SDS-PAGE loading buffer (Invitrogen) containing Tris-base. Protein extracts were directly resolved on Tris-acetate 3–8% polyacrylamide NuPAGE gels (Invitrogen). Proteins were transferred to a nitrocellulose Hybond-C membrane (Invitrogen). The membrane was blocked in PBS-T milk 5% and probed by using anti-Flag (Sigma F1804) antibody (1:5,000 dilution), anti Cdc2 (Santa-Cruz sc-53) antibody (1,1000 dilution), anti-tubulin alpha T5168 (Sigma).

### Recombinant MBP-Eme1-Mus81-6-His production and purification

The cDNA of *eme1* and *mus81* were subcloned into the pMBP-parallel1 and pCDFDuet-1 plasmids respectively using in-fusion (Takara) cloning system. A 6-His tag was inserted in frame with mus81 ORF for C-terminal of the protein. Plasmids were co-transformed in Rosetta (DE3) pLysS cells. The expression of MBP-Eme1 and Mus81-6His was carried by growing the cells into auto-induced media (Formedium) at 37°C. Cells were harvest at 4°C, resuspended in PBS 1X and kept at -20°C. Lysis buffer 2X (100mM Tris-HCl, pH = 8.0, 300mM NaCl, 20% glycerol, 0,2% NP-40, 2mM PMSF, 2mM ß-Mercaptoethanol, protease inhibitor cocktail complete EDTA-free (Roche), 10 mg/mL lysozyme, 20mM imidazole) was added to lysed cells before incubation for 20 min at 4°C. The lysate was cleared by centrifugation before incubation on Ni^2+^ agarose beads (Qiagen) for 2h at 4°C. The beads were washed with 5 volumes of lysis buffer 1X and eluted with lysis buffer 1X supplemented with 250mM imidazole. Eluted complexes were incubated with amylose beads (NEB) for 2h at 4°C. The beads were washed 3 times in lysis buffer 1X and twice in kinase buffer (25mM HEPES-KOH, pH = 7.5, 50mMKCl, 10mM MgCl_2_, 10mM MnCl_2_, 2% glycerol, 0,1% NP-40, 50mM NaF, 1mM Na_3_VO_4_, 50mM ß-glycerophosphate, 1mM DTT). Complexes were eluted in kinase buffer supplemented with 10mM maltose. Proteins were aliquoted, snap-frozen in liquid nitrogen and stored at -80°C for long term storage.

### GFP-Rad3^ATR^ production and *in vitro* kinase assay

GFP-Rad3^ATR^ was transiently expressed from the full-length nmt41 promoter from *cds1Δ chk1Δ rad3Δ* cells treated 2h with bleomycin. Cells pellets were disrupted using a Ball Mill (Retsch) in presence of liquid Nitrogen. Resulting powder was resuspended in 2 volumes/weight of lysis buffer (50mM Tris-HCl, pH = 8.0, 500mM NaCl, 10% glycerol, 1% NP-40, 50mM NaF, 1mM Na_3_VO_4_, 50mM ß-glycerophosphate (Sigma), 2mM PMSF, 1mM DTT, protease inhibitor cocktail complete EDTA free (Roche)). Lysates were cleared by centrifugation before incubation on GFP-TRAP agarose beads (Chromotek) at 4°C for 1h. Beads were washed three times with lysis buffer and twice with kinase buffer (25 mM HEPES-KOH, pH = 7.5, 50mM KCl, 10mM MgCl_2_, 10mM MnCl_2_, 2% glycerol, 0,1% NP-40, 50mM NaF, 1mM Na_3_VO_4_, 50mM β-glycerophosphate, 1mM DTT). GFP-Rad3^ATR^ was kept attached on beads for the following kinase assays.

GFP-TRAP-bound GFP-Rad3^ATR^ was resuspended in kinase buffer supplemented 100 μM of cold ATP before addition of 10 μCi γ^32^P-ATP and substrates. After 30 min at 30°C, reactions were stopped by the addition of 15 μL of 4X SDS sample buffer. Samples were denatured and resolved by SDS-PAGE electrophoresis. Following Coomassie staining, gel was dried and expose with phosphorimager.

### *In vitro* nuclease assay

TAP-Eme1 (2xProtA-TEVsite-2xFlag-Eme1) was affinity purified and used in nuclease assays on X12 mobile HJ as previously described [28]. Briefly, cell pellets were resuspended in 1 volume to weight lysis buffer (50mM Tris-HCl, pH = 8.0, 150 mM NaCl, 10% glycerol, 0,1% NP-40, 50mM NaF, 50mM β-glycerophosphate (Sigma), 2mM PMSF, protease inhibitor cocktail complete EDTA free (Roche)). Cells were subjected to mechanical lysis using a Ball Mill (MM400 Retsch). For this, usually, 4 to 5 ml of the cell suspension were poured into grinding chambers precooled in liquid nitrogen. The frozen cell pellet was disrupted by 2 agitation runs at 30hz. The resulting powder was resuspended in another 1 volume to weight of lysis buffer and centrifuged. Clear supernatant was loaded onto IgG sepharose beads (Cytiva) for 2h at 4°C (20 μl packed beads used for 4 ml of lysate). After extensive washes, proteins were eluted in presence of 60 μl of AcTEV protease for 1h at RT. In order to determine the relative amount of Mus81-Eme1 between different samples, 3 μl of each TEV eluate was treated with phosphatase before SDS-PAGE and Western blot analysis in order to collapse the Eme1 signal into a single band of dephosphorylated Eme1. The relative intensity of the dephosphorylated Eme1 band was quantified for each sample using the ImageLab software. Dilution folds were calculated and used to bring the concentration of each sample down to that of the least concentrated sample. 3 μl of each normalized sample was used in nuclease assays with ^32^P-labelled DNA substrates as previously described [28].

TCA (Sigma T6399)

Glass beads (Sigma G8772)

Calcofluor (Sigma 18909)

S-(+)-Camptothecin (Sigma C9911)

Bleomycin (Calbiochem 9041-93-4)

Hydroxyurea (Sigma H8627)

Protease inhibitor Cocktail EDTA-free (Roche 11873580001)

Anti Flag (Sigma F1804)

Anti Cdc2 (Santa-Cruz sc-53)

Anti tubulin (Sigma T5168)

GFP-TRAP magnetic-agarose (Chromotek gtma-20)

Ni-NTA agarose beads (Qiagen 70666–3)

Amylose resin (NEB E8021S)

Phosphatase (NEB P0753S)

IgG Sepharose 6 Fast Flow (Cytiva 17-0969-01)

AcTEV (Invitrogen 12575015)

## Supporting information

S1 Fig The Rad3^ATR^-consensus sites are widespread throughout Eme1 sequence and were subdivided in three clusters (Upper panel). All Rad3^ATR^-consensus sites are mutated in Alanine to generate eme1^8AQ^ mutant (Lower panel).
(PDF)

S2 Fig A- Exponentially growing cultures of *eme1^8AQ^*, *rqh1Δ* and *eme1^8AQ rqh1Δ^* cells were heated-fixed and observed by fluorescent-microscopy using DAPI staining. Cells were classified based on their morphologies. Small G2 cells (Type 1), elongated bi-nucleated and/or septated cells (Type 2) and sick cells (Type 3). B- Five-fold dilutions of cells with the indicated genotype were plated on medium supplemented or not with the indicated concentrations of CPT, HU and MMS followed by incubation at 30°C. C- Tetrad analysis of an eme1^cluster1*^, eme1^cluster2*^ and eme1^cluster3*^ x *rqh1Δ* mating, germinated at 30°C. To assess the impact of introducing the cluster mutations (C*) in an rqh1Δ background, the ratio of the colony diameter of the double mutants C* *rqh1Δ* mutants over that of the *rqh1Δ* mutant were calculated for each tetrad and plotted on the graph below the tetrads.
(PDF)

S3 Fig A- Western Blot on the indicated TEV-eluates after phosphatase treatment and normalization of their relative concentration, as described in Materials and Methods. B- Neutral PAGE showing ^32^P-labeled (red dot) HJs incubated for the indicated times with Mus81–Eme1 complexes recovered from untreated or 40 μM CPT–treated *TAP-eme1* (“wild type”) cells as described in Materials and Methods. Comparable amounts of TEV eluates were used in each reaction after normalization of their relative concentration (see Materials and Methods and **S3A Fig**). Graphs below the autoradiographs represents the quantification of product formation, as a percentage of total radiolabeled DNA, for each experiment. Note: Schematic depicting the migration profile of uncleaved and cleaved radiolabelled HJs. Yellow dotted boxes represent areas of the gel used for quantification purposes and calculation of the percentage of cleavage product. The “Bckg” box corresponds to background signal that was subtracted from the “Uncut” and “Cut” signals. C- Same as A- but with Mus81–Eme1 from untreated or 40 μM CPT–treated *TAP-eme1 rad3Δ* cells. D- Same as A- but with Mus81–Eme1 from untreated or 40 μM CPT–treated *TAP-eme1 eme1*^*8AQ*^ cells. E- Same as A- but with Mus81–Eme1 from untreated or 40 μM CPT–treated *TAP-eme1 chk1Δ* cells. Note: TAP- = 2xProtA-TEVsite-2xFlag-.
(PDF)

S4 Fig A- Cells from exponentially growing cultures of the indicated genotypes were heated-fixed and observed by fluorescent-microscopy using DAPI staining (same as S2), counted and sorted depending on their morphologies. B- Five-fold dilutions of cells with the indicated genotype were plated on medium supplemented or not with the indicated concentrations of CPT followed by incubation at 30°C.
(PDF)

S5 Fig Same as for S3B Fig but for Mus81-Eme1^SIM1*+SIM2*^ complex from untreated or 40 μM CPT–treated *eme1*^SIM1*+SIM2*^ cells. (PDF)

S6 Fig Recombinant Mus81 was co-produced in *E*. *coli* with either wild-type Eme1 or Eme1^SIM1*+SIM2*^ and purified as described in Fig 2A. Rad3^ATR^ *in vitro* kinase assays were carried out on the corresponding recombinant Mu81-Eme1 and Mus81-Eme1^SIM1*+SIM2*^ complexes. A representative autoradiography of ^32^P labeled Eme1 is shown. Relative band intensity of phosphorylated Eme1 (n = 3) shows that Eme1^SIM1*+SIM2*^ is efficiently phosphorylated *in vitro* by Rad3^ATR^.
(PDF)

S7 Fig Western blot detection of TAP-Eme1 from untreated or 40 μM CPT treated WT, *pli1Δ*, *nse2*^*SA*^, *pli1Δ nse2*^*SA*^ and *pmt3Δ* cells. Western blot using an antibody raised against the Flag tag of TAP-Eme1. Tubulin is used as a loading control. Note: TAP- = 2xProtA-TEVsite-2xFlag-.
(PDF)

S1 Table Complete list of S. pombe strains used in this study. (XLSX)
