# Supplementary figures and images for: Regulation of Mus81-Eme1 structure-specific endonuclease by Eme1 SUMO-binding and Rad3ATR kinase is essential in the absence of Rqh1BLM helicase
Source: PLoS Genet. 2022 Apr 22;18(4):e1010165. doi: 10.1371/journal.pgen.1010165 (PMC9032445; doi:10.1371/journal.pgen.1010165)

Supplementary Figure 1

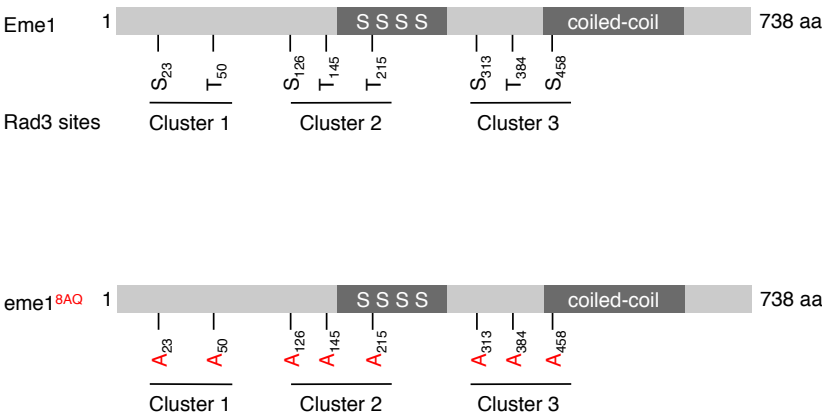

Supplement: S1 Fig — All Rad3ATR-consensus sites are mutated in Alanine to generate eme18AQ mutant (Lower panel). (PDF) [file pgen.1010165.s001.pdf]

Supplementary Figure 2

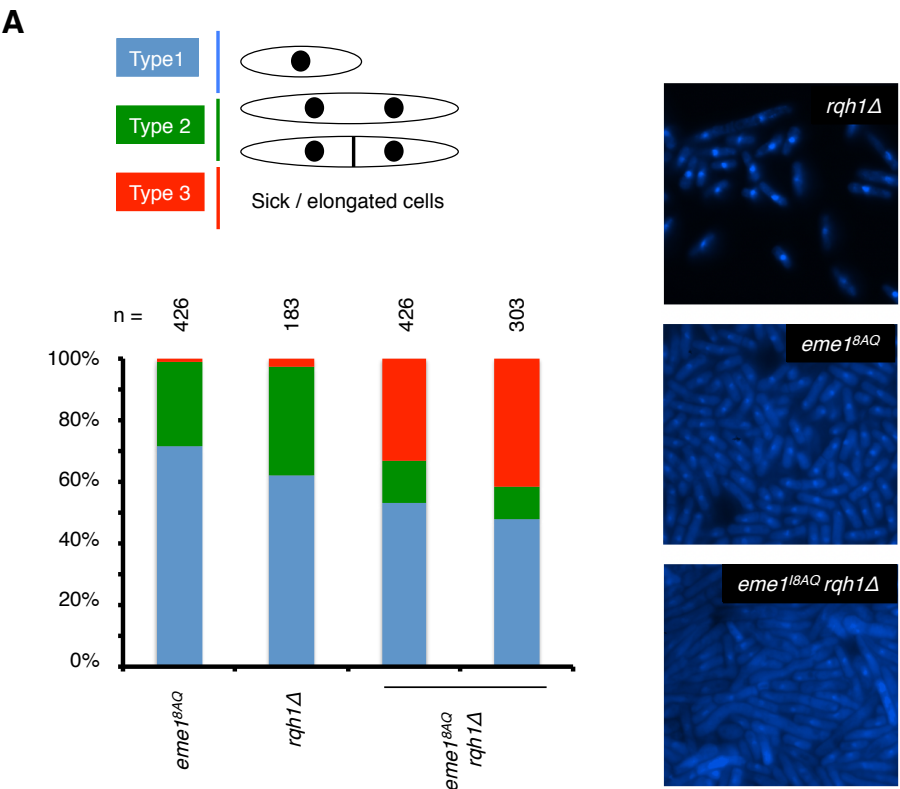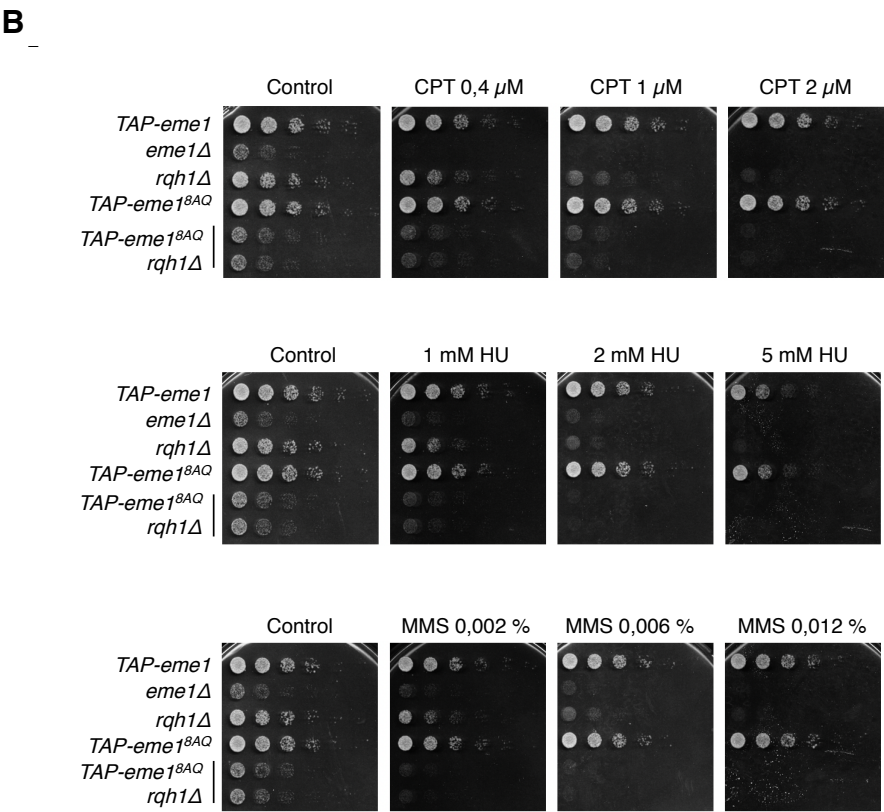

Supplementary Figure 2

C

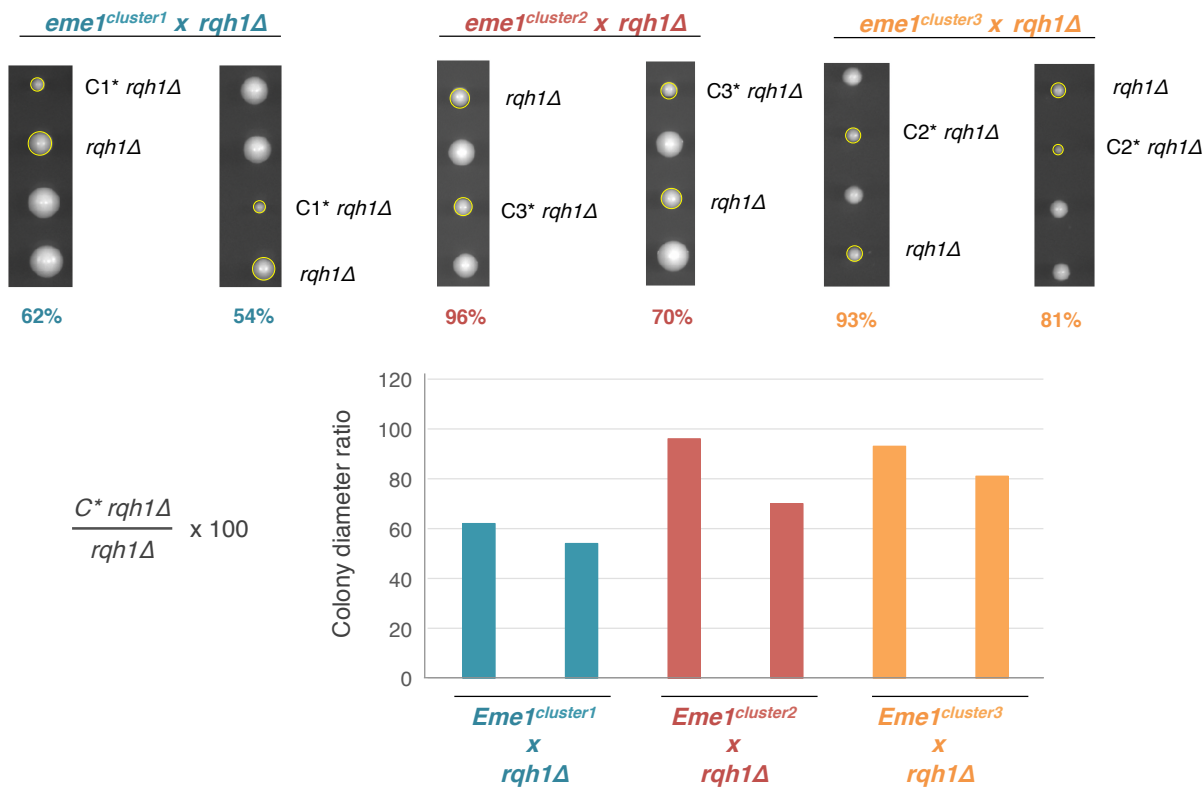

Supplement: S2 Fig — A- Exponentially growing cultures of eme18AQ, rqh1Δ and eme18AQ rqh1Δ cells were heated-fixed and observed by fluorescent-microscopy using DAPI staining. Cells were classified based on their morphologies. Small G2 cells (Type 1), elongated bi-nucleated and/or septated cells (Type 2) and sick cells (Type 3). B- Five-fold dilutions of cells with the indicated genotype were plated on medium supplemented or not with the indicated concentrations of CPT, HU and MMS followed by incubation at 30°C. C- Tetrad analysis of an eme1cluster1*, eme1cluster2* and eme1cluster3* x rqh1Δ mating, germinated at 30°C. To assess the impact of introducing the cluster mutations (C*) in an rqh1Δ background, the ratio of the colony diameter of the double mutants C* rqh1Δ mutants over that of the rqh1Δ mutant were calculated for each tetrad and plotted on the graph below the tetrads. (PDF) [file pgen.1010165.s002.pdf]

Supplementary Figure 3

A

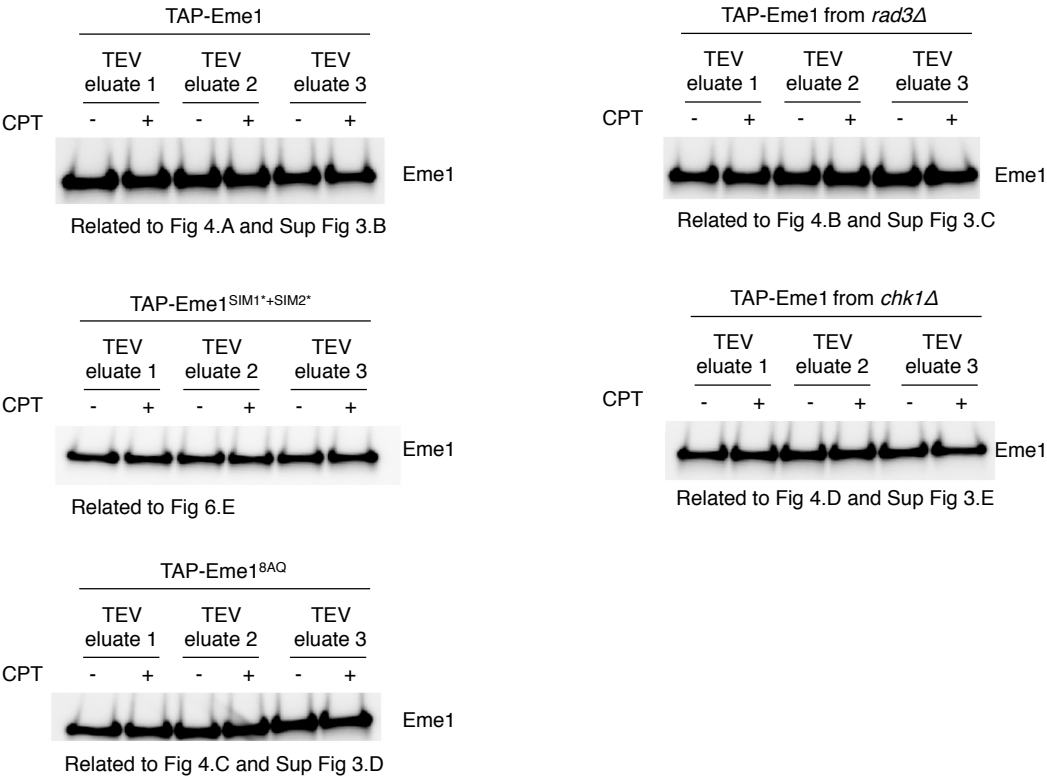

Supplementary Figure 3

B

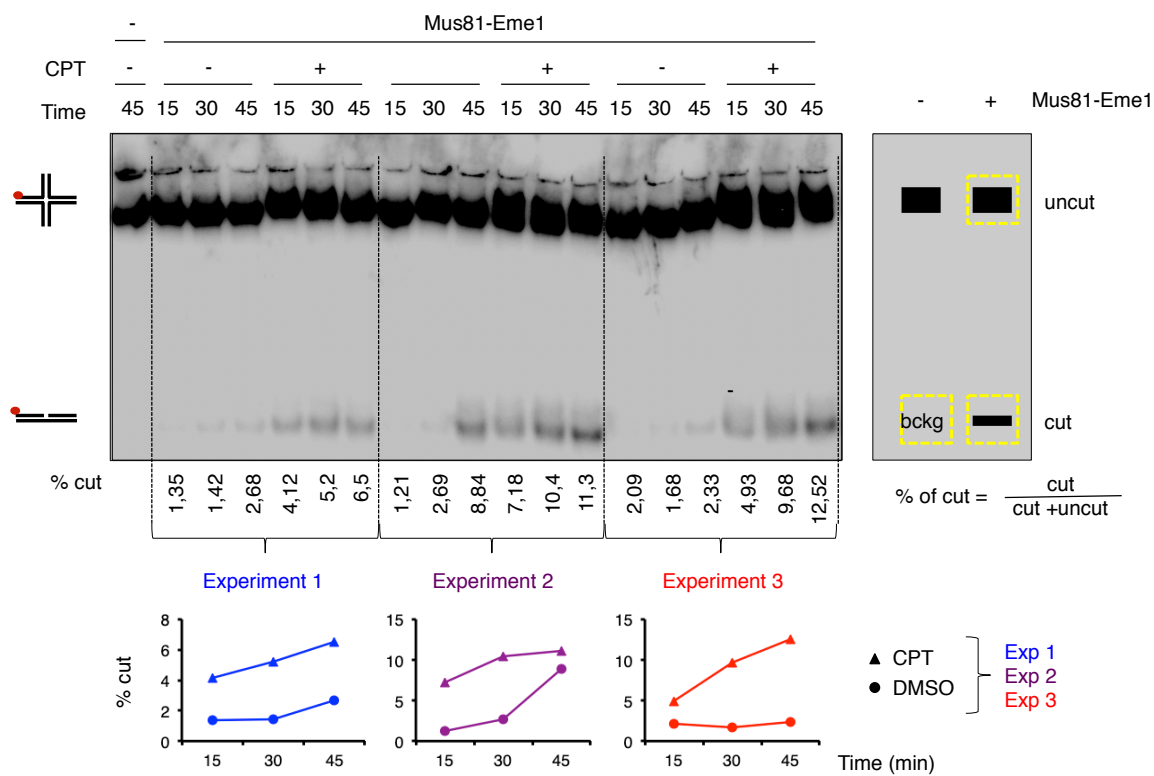

C

Supplementary Figure 3

D

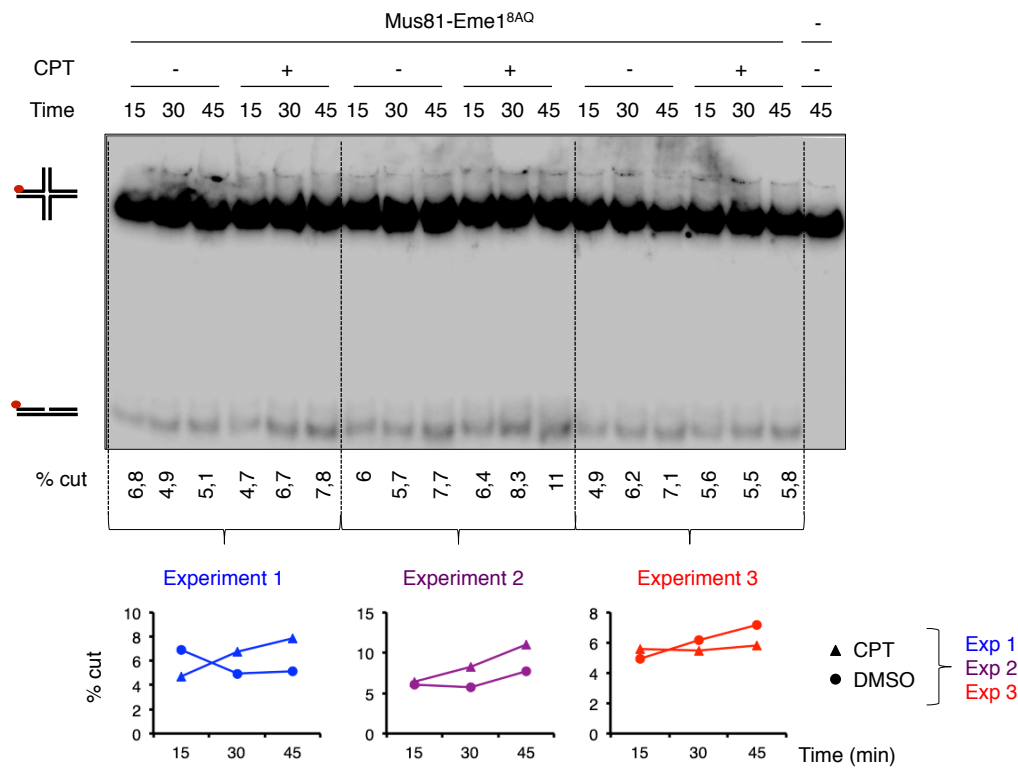

E

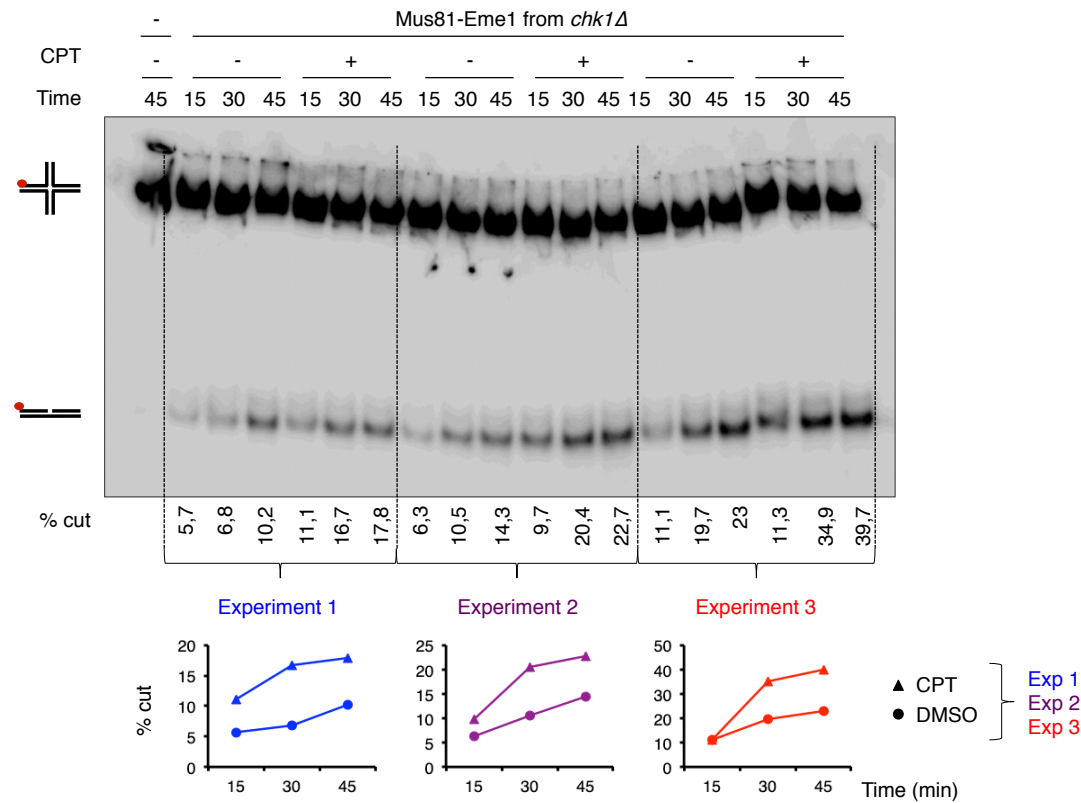

Supplement: S3 Fig — B- Neutral PAGE showing 32P-labeled (red dot) HJs incubated for the indicated times with Mus81–Eme1 complexes recovered from untreated or 40 μM CPT–treated TAP-eme1 (“wild type”) cells as described in Materials and Methods. Comparable amounts of TEV eluates were used in each reaction after normalization of their relative concentration (see Materials and Methods and S3A Fig). Graphs below the autoradiographs represents the quantification of product formation, as a percentage of total radiolabeled DNA, for each experiment. Note: Schematic depicting the migration profile of uncleaved and cleaved radiolabelled HJs. Yellow dotted boxes represent areas of the gel used for quantification purposes and calculation of the percentage of cleavage product. The “Bckg” box corresponds to background signal that was subtracted from the “Uncut” and “Cut” signals. C- Same as A- but with Mus81–Eme1 from untreated or 40 μM CPT–treated TAP-eme1 rad3Δ cells. D- Same as A- but with Mus81–Eme1 from untreated or 40 μM CPT–treated TAP-eme1 eme18AQ cells. E- Same as A- but with Mus81–Eme1 from untreated or 40 μM CPT–treated TAP-eme1 chk1Δ cells. Note: TAP- = 2xProtA-TEVsite-2xFlag-. (PDF) [file pgen.1010165.s003.pdf]

Supplementary Figure 4

A

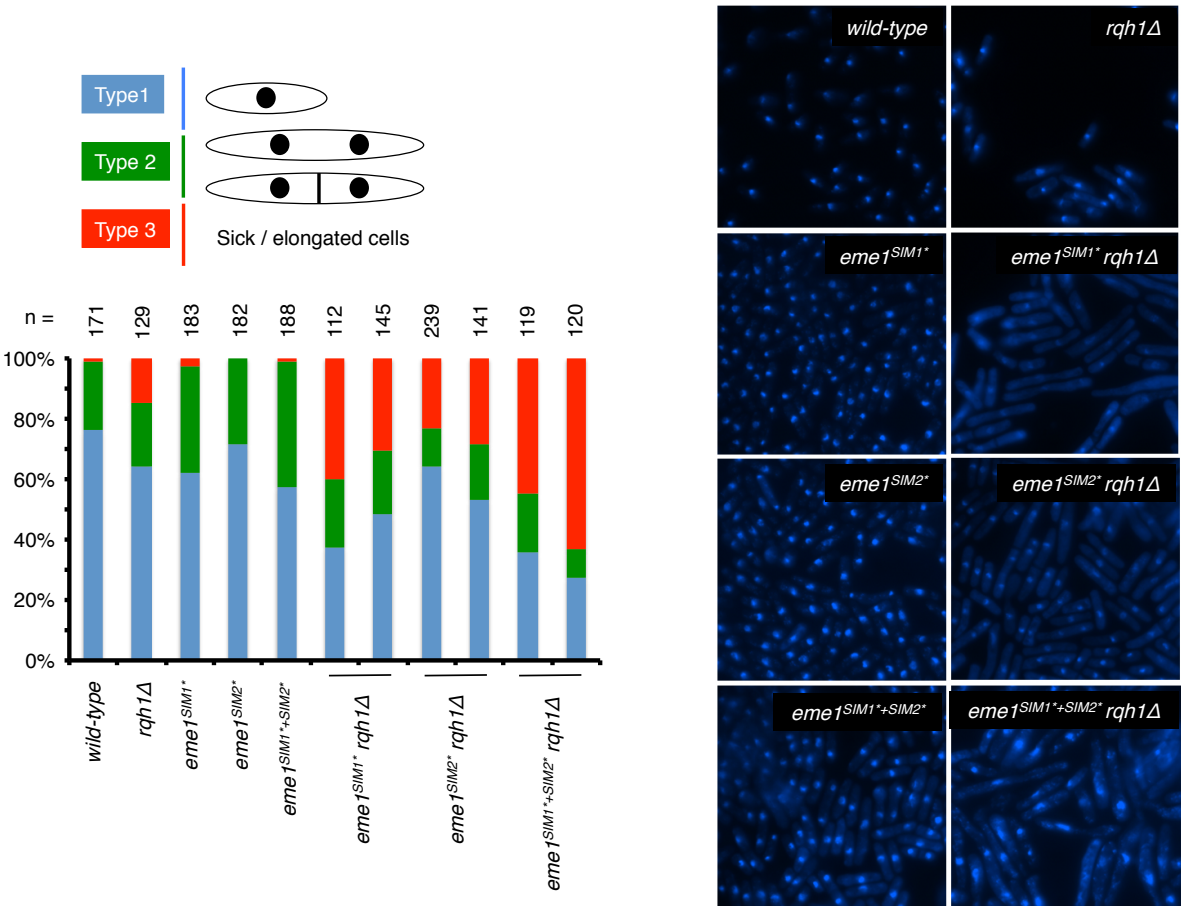

B

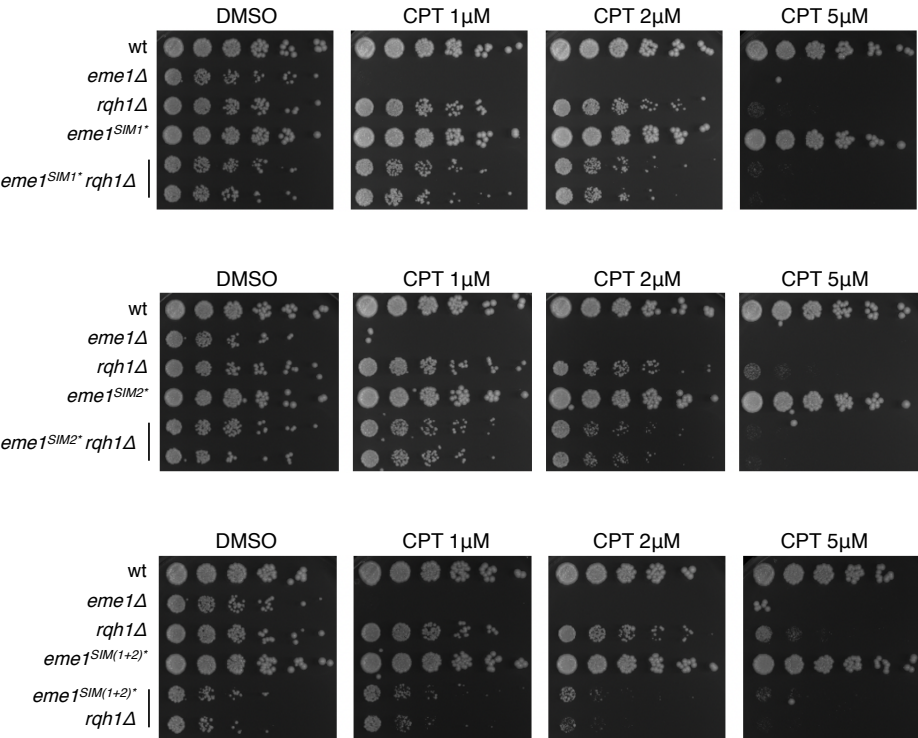

Supplement: S4 Fig — B- Five-fold dilutions of cells with the indicated genotype were plated on medium supplemented or not with the indicated concentrations of CPT followed by incubation at 30°C. (PDF) [file pgen.1010165.s004.pdf]

Supplementary Figure 5

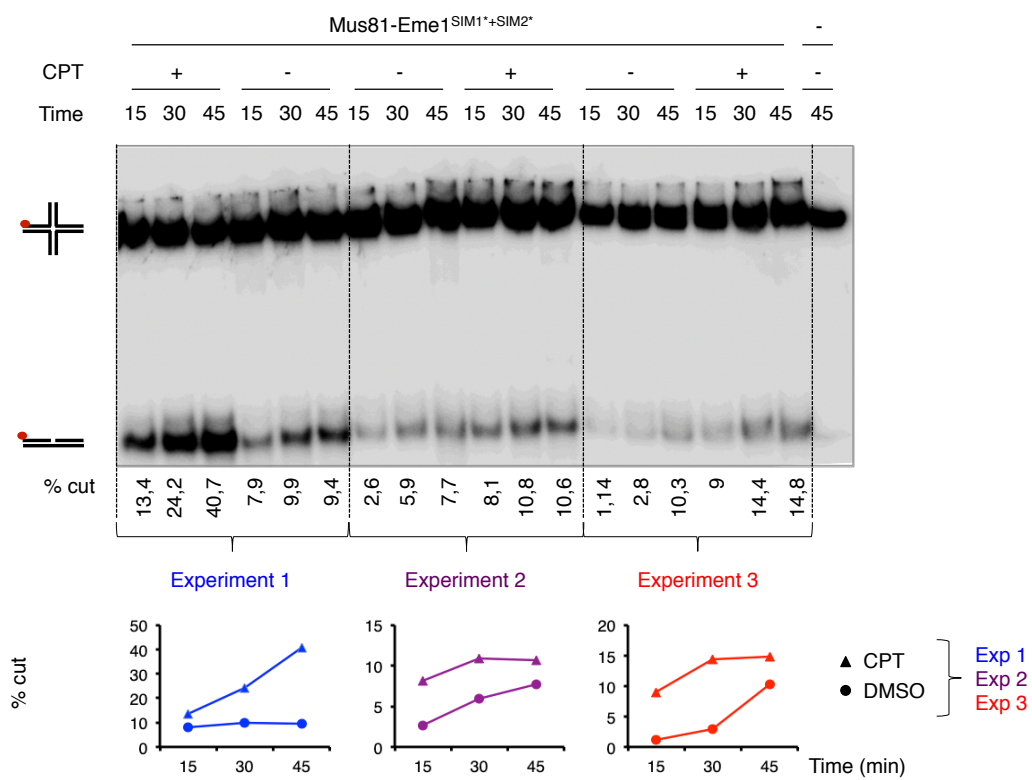

Supplement: S5 Fig — (PDF) [file pgen.1010165.s005.pdf]

Supplementary Figure 6

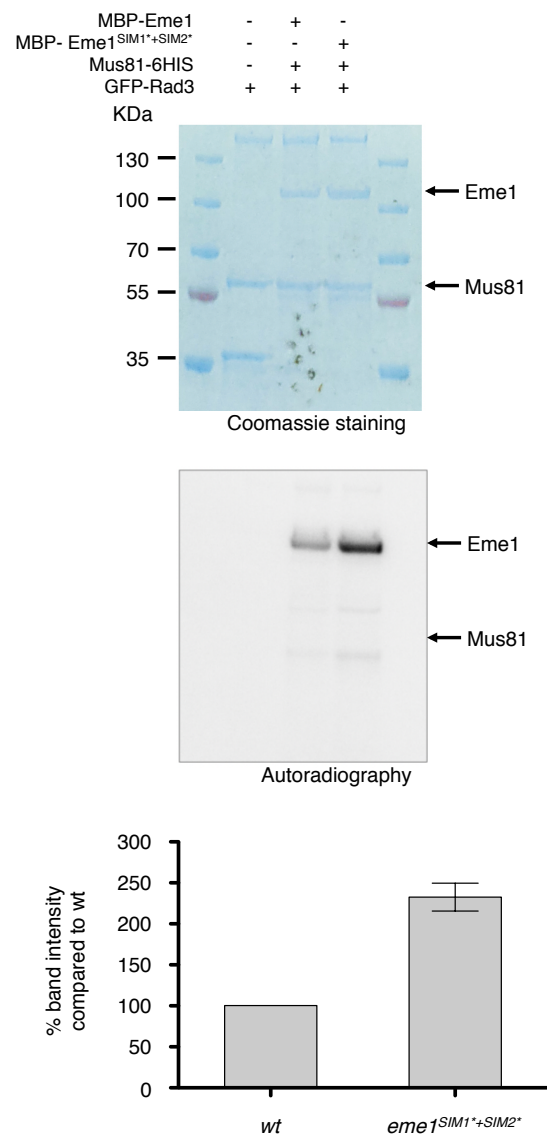

Supplement: S6 Fig — Rad3ATR in vitro kinase assays were carried out on the corresponding recombinant Mu81-Eme1 and Mus81-Eme1SIM1*+SIM2* complexes. A representative autoradiography of 32P labeled Eme1 is shown. Relative band intensity of phosphorylated Eme1 (n = 3) shows that Eme1SIM1*+SIM2* is efficiently phosphorylated in vitro by Rad3ATR. (PDF) [file pgen.1010165.s006.pdf]

Supplementary Figure 7

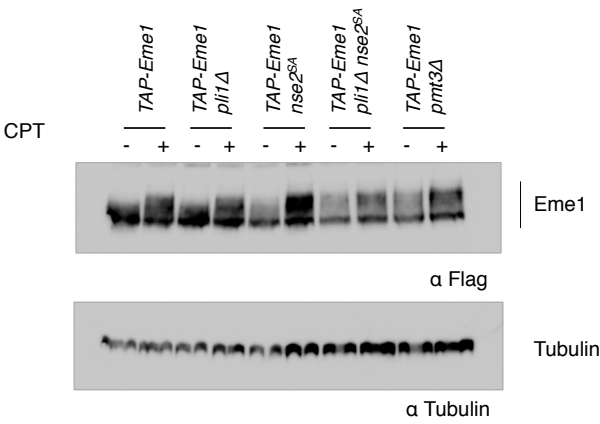

Supplement: S7 Fig — Western blot using an antibody raised against the Flag tag of TAP-Eme1. Tubulin is used as a loading control. Note: TAP- = 2xProtA-TEVsite-2xFlag-. (PDF) [file pgen.1010165.s007.pdf]
